# Qishiwei Zhenzhu Pills Protect Against Cerebral Ischemia via the P53/Cytochrome C/Apoptotic Protease Activating Factor 1‐Mediated Mitochondrial Apoptosis Pathway

**DOI:** 10.1111/cns.70476

**Published:** 2025-06-26

**Authors:** Yinglian Song, Guili Song, Lame Lizhen, Yan Liang, Mengtian Han, Yichu Yang, Qiaoqiao Feng, Yi Li, Jingwen Zhang, Min Xu, Yongzhong Zeweng, Miao Jiang, Zhang Wang

**Affiliations:** ^1^ College of Pharmacy Chengdu University of Traditional Chinese Medicine Chengdu China; ^2^ State Key Laboratory of Southwestern Chinese Medicine Resources Chengdu University of Traditional Chinese Medicine Chengdu China; ^3^ College of Ethnomedicine Chengdu University of Traditional Chinese Medicine Chengdu China

**Keywords:** cerebral ischemia, components that enter the bloodstream, metabolomics, mitochondrial apoptosis pathway, Qishiwei Zhenzhu pills, UPLC‐Q‐TOF‐MS

## Abstract

**Background:**

Qishiwei Zhenzhu pills (QSWZZP, Tibetan name: 
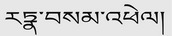
) originated from Ershiwuwei Zhenzhu mother pills in the eighth century AD and are currently included in the *Chinese Pharmacopoeia* (2020). QSWZZP calm the mind, activate the medians, regulate qi, and harmonize the blood. QSWZZP have significant therapeutic effects in cerebral ischemia. However, QSWZZP's complex and diverse multi‐herb chemical composition has presented challenges in identifying their active ingredients, which have hitherto remained unclarified. Therefore, the present study focusses on the identification of QSWZZP's active ingredients and their therapeutic mechanisms.

**Aims:**

To analyze the distribution of QSWZZP's components in the blood and tissues for the treatment of cerebral ischemia, to identify the biomarkers after drug intervention, and to explore the mechanism of action of QSWZZP in terms of the mitochondrial apoptosis pathway.

**Methods:**

Ultra‐high performance liquid chromatography with quadrupole time‐of‐flight mass spectrometry was used for qualitative and metabolomic analysis of the distribution of QSWZZP's components in the blood and tissues. A middle cerebral artery occlusion (MCAO) rat model was used to evaluate and measure neurobehavioral changes, the ratio of cerebral infarction, the rate of apoptosis‐positive cells in the brain tissue, and the pathological changes in the cerebral cortex, hippocampus, and diencephalon. mRNA, protein, and fluorescence expressions of apoptosis‐inducing factor (AIF), P53, cytochrome C (Cyt C), apoptotic protease activating factor‐1 (APAF‐1), cleaved caspase‐8, B‐cell lymphoma‐extra‐large, and N‐myc downstream‐regulated gene family member 4 (NDRG4) were determined in the rat brain tissues via real‐time polymerase chain reaction, western blotting, and immunofluorescence. In addition, molecular docking was used to screen the active components of QSWZZP against mitochondria‐mediated apoptosis.

**Results:**

Thirty‐three new compounds were identified, including 13 triterpenoids and nine flavonoids. Among them, 15 blood‐entry, 21 urine‐entry, three brain‐entry, seven liver‐entry, and four kidney‐entry components were identified. QSWZZP significantly improved neurobehavioral abnormalities and reduced the cerebral infarction rate in the MCAO rats by significantly decreasing AIF, P53, Cyt C, APAF‐1, and cleaved caspase‐8 mRNA expressions and Cyt C and APAF‐1 protein expressions, as well as increasing NDRG4 protein expression in the rat brain tissues. Molecular docking revealed that the active ingredients of QSWZZP against mitochondrial‐mediated apoptosis were arjunic acid, cholic acid, and phyllaemblic acid.

**Conclusion:**

In this study, 45 components of QSWZZP were qualitatively analyzed, and the metabolic pathways of the related products were clarified. The potential treatment mechanism of QSWZZP may be related to the modulation of the folate biosynthesis metabolic and P53/Cyt C/APAF‐1‐mediated mitochondrial apoptosis pathways. This study may serve as the foundation for subsequent pharmacokinetic experiments and analysis of the material basis of the drug effect of QSWZZP in cerebral ischemia treatment.

## Graphical abstract

Ultra‐high performance liquid chromatography with quadrupole time‐of‐flight mass spectrometry was used for qualitative and metabolomic analysis of the distribution of Qishiwei Zhenzhu pills’ (QSWZZP, Tibetan name: རཏྣ་བསམ་འཕེལ།) components in the blood and tissues. A middle cerebral artery occlusion (MCAO) rat model was used to evaluate and measure neurobehavioral changes, the ratio of cerebral infarction, the rate of apoptosis‐positive cells in the brain tissue, and the pathological changes in the cerebral cortex, hippocampus, and diencephalon. mRNA, protein, and fluorescence expressions of apoptosis‐inducing factor (AIF), P53, cytochrome C (Cyt C), apoptotic protease activating factor‐1 (APAF‐1), cleaved caspase‐8, B‐cell lymphoma‐extra‐large, and N‐myc downstream‐regulated gene family member 4 (NDRG4) were determined in the rat brain tissues via real‐time polymerase chain reaction, western blotting, and immunofluorescence. In addition, molecular docking was used to screen the active components of QSWZZP against mitochondria‐mediated apoptosis.

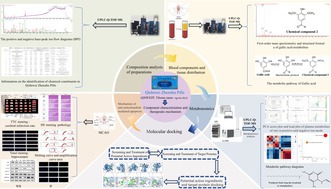

**Abbreviations**
ADP‐glucose: adenosine diphosphoglucose
AIF: apoptosis‐inducing factor
APAF: apoptotic protease activating factor‐1
AppppA: diadenosine tetraphosphate
ATP: adenosine triphosphate
BAX: B‐cell lymphoma protein 2‐associated X
BCL‐2: B‐cell lymphoma protein 2
BCL‐XL: B‐cell lymphoma‐extra‐large
BPI: base peak ion
CCA: common carotid artery
CDP‐glucose: cytidine diphosphoglucose
Cyt C: cytochrome C
dATP: deoxyadenosine triphosphate
ECA: EXTERNAL carotid artery
ESI: electrospray ionization
GC–MS: Gas chromatography
Glc: glucose
Glu A: D‐glucuronic acid
GSH‐PX: glutathione peroxidase
HE: hematoxylin and eosin
HES: hairy and enhancer of split
HMDB: Human Metabolome Database
HPLC: high‐performance liquid chromatography
ICA: internal carotid artery
ICP: inductively coupled plasma
IF: immunofluorescence
IL: interleukin
KEGG: Kyoto Encyclopaedia of Genes and Genomes
LIBS: laser induced breakdown spectroscopy
MCAO: middle cerebral artery occlusion
MDA: malondialdehyde
MS: mass spectrometry
NDRG4: N‐myc downstream‐regulated gene family member 4
NOTCH 1: neurogenic locus notch homolog protein 1
OD: optical density
OPLS‐DA: orthogonal partial least squares discriminant analysis
PBS: phosphate‐buffered saline
PC: phosphatidylcholine
PCA: principal component analysis
PE: phosphatidylethanolamine
PG: prostaglandin
PUMA: P53 upregulated modulator of apoptosis
QSWZZP: Qishiwei Zhenzhu pills
RDA: Retro Diels‐Alder
SOD: superoxide dismutase
TUNEL: terminal deoxynucleotidyl transferase deoxyuridine triphosphate nick end labelling
UPLC‐Q‐TOF‐MS: ultra‐high‐pressure liquid chromatography coupled with electrospray time‐of‐flight tandem mass spectrometry
WB: western blotting

## 1 Introduction

Cerebral ischemia is defined as insufficient blood supply to a specific area of the brain due to obstructed blood vessels. The basic treatment strategy, via intravenous thrombolysis and endovascular therapy, focuses on promptly restoring the blood supply to the ischemic area [1]. However, intravenous thrombolysis has a narrow timeframe and tends to induce hemorrhage, exacerbating neurological damage [1], thus highlighting the need for discovering neuroprotective drugs against acute ischemic stroke [1].

Tibetan medicine is an important part of traditional Chinese medicine. Tibetan medicine experts have, over several dynasties, established a relatively perfect theoretical system through continuous practice and research [2]. In Tibetan medicine theory, cerebral ischemia is a type of ‘Baimai’ disease, or *Long zhi bu* disease. *Zhi bu* refers to fragility and decline. In Tibetan medicine theory, the disease is a ‘Baimai’ dysfunction‐induced encephalopathy due to heat evil, plague, pathogen invasion, external force injury, and *Long* imbalance [3, 4]. The disease typically manifests as limb movement disorders, language dysfunction, mouth and eye deviation, and consciousness. Tibetan medicine treatment for Baimai disease includes both overall and specific treatments to restore and improve Baimai function [3, 5]. Qishiwei Zhenzhu pills (QSWZZP. Tibetan name: 
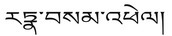
), a well‐known and valued Tibetan medicine, originated from *Ershiwuwei Zhenhua* mother pills in the eighth century AD [6, 7]. It was recorded in the 18th century seminal classic, *Collection of Ten Thousand Medical Prescriptions* [8]. Furthermore, QSWZZP were approved by the Chinese Ministry of Health as a national protected variety of Traditional Chinese Medicine in 1997 and were included in the *Chinese Pharmacopoeia* (2020 edition). The recipe of QSWZZP contains > 70 medicinal materials, such as *Dalbergia odorifera* T. Chen., *Styrax benzoin* Dryand., *Nepeta hemsleyana* Oliv. Ex Prain., *Lagotis brachystachya* Maxim., *Aquilaria sinensis* (Lour.) Spreng., *Santalum album* L., *Phyllanthus emblica* L., *Piper longum* L., *Codonopsis xizangensis* D. Y. Hong., *Syzygium aromaticum* (L.) Merr. & L. M. Perry., *Amomum compactum* Soland ex Maton., *Nigella glandulifera* Freyn & Sint, *Myristica fragrans* Houtt., and *Schizostachyum chinense* Rendl., among others [7, 9]. Most commonly used for the treatment of various acute and chronic cerebrovascular and nervous system diseases in Tibetan medicine [10, 11], QSWZZP tranquilize the mind, calm the nerves, activate the meridians, and regulate qi and blood in Tibetan medicine theory [10, 12, 13, 14]. Traditionally, QSWZZP are used to treat ‘Hei’ and Baimai diseases, *Long* blood disorder, stroke, paralysis, hemiplegia, epilepsy, and cerebral hemorrhage [15, 16, 17]. QSWZZP have significant benefits particularly in the treatment of cerebral ischemia [18, 19, 20].

Several researchers in China, including the authors of the present study, have conducted numerous studies on the clinical application, safety [21, 22], dose‐effect, ingredients, and mechanism of action of QSWZZP. Luo et al.'s study included 208 patients (138 and 70 cases of ischemic and hemorrhagic stroke, respectively) out of 4563 patients with typical sequelae of cerebral stroke. They used mainly QSWZZP for treatment and observation, achieving total effective rates of 86.2% and 80%, respectively [23]. Du et al.'s clinical safety evaluation study assessed the composition of QSWZZP and monitored and analyzed the content and metabolism of three different heavy metals in human blood, urine, and feces before the drug administration, during 15 days after drug administration, and 15 days after drug cessation [24]. Liang et al. found that the optimal effect time point for the treatment of cerebral ischemia–reperfusion injury (CI/RI) in rats was 48 h, with a maximum effective dose of 66.68 mg/kg of QSWZZP. The improvement mechanism may be related to increased superoxide dismutase (SOD) and reduced malondialdehyde (MDA) levels [25].

Wu et al.'s qualitative study on QSWZZP's mineral composition used laser‐induced breakdown spectroscopy (LIBS), and they found > 10 elements, including minerals such as magnesium, calcium, sodium, iron, aluminum, silicon, and potassium and heavy metals such as mercury, lead, and gold [26]. Li et al.'s analyses on the long‐term accumulation and distribution of QSWZZP mineral elements in vivo found that most of these elements were not accumulated in animal tissues and organs (e.g., heart, liver, and kidneys) and that they were mainly metabolized by the liver and kidneys and subsequently excreted [27]. Yan et al. evaluated the chemical constituents in the serum of mice post‐gavage of QSWZZP using high‐performance liquid chromatography–tandem mass spectrometry (HPLC–MS/MS), and identified 44 active ingredients, including 13 possible prototype drug components [28]. Song et al. found low levels of 18 QSWZZP elements, including lithium, beryllium, scandium, vanadium, chromium, manganese, cobalt, lead, gold, and mercury, accumulated in the blood, brain, liver, and kidneys and clarified that the elements were mainly excreted through feces. Moreover, the significant changes induced by lithium, chromium, and cadmium in the brain suggested their possible material basis in cerebral ischemia treatment [29]. Xu used ultra‐high performance liquid chromatography with quadrupole time‐of‐flight mass spectrometry (UPLC‐Q‐TOF‐MS) to qualitatively analyze 42 QSWZZP components, including corilagin, agarotetrol, and ellagic acid, and HPLC–MS to further quantitatively analyze 16 QSWZZP components, including corilagin, quercetin, and agarotetrol [30]. While Xu's study explored the volatile blood‐entry components of QSWZZP, Liu et al.'s study used LC‐MS to analyze and identify the other components that were distributed in the blood and tissue to help determine the material basis of the medicinal effect of QSWZZP [31]. *Crocus sativus* L. and *Salvia miliorrhiza* Bunge. are some of the main components of QSWZZP, which have been reported to significantly improve cerebral ischemia [32, 33, 34, 35].

Cerebral ischemia‐induced mitochondrial apoptosis further aggravates brain tissue injury. Therefore, the inhibition of mitochondrial apoptosis has become a vital target in cerebral ischemia treatment. Sun et al. investigated the mitochondrial apoptosis inhibitory mechanism of propofol in protecting against cerebral ischemia and revealed that the drug reduced apoptosis‐inducing factor (AIF) and cytochrome C (Cyt C) expression in rat brain tissue [36]. Liang et al. used UPLC‐Q‐TOF‐MS and ultra‐high‐performance liquid chromatography with triple quadrupole mass spectrometry (HPLC‐QQQ‐MS) to qualitatively and quantitatively analyze QSWZZP and revealed that the protective effect of QSWZZP in cerebral ischemia, from the perspective of inflammation and the apoptosis pathway, may be related to the down‐regulation of the apoptosis factor, caspase‐3 [9]. Zhang demonstrated that mullein isoflavones could prevent oxygen–glucose deprivation‐induced cellular damage in PC12 cells via regulation of the Cyt C/apoptotic peptidase activating factor 1 (APAF‐1) mitochondrial apoptosis pathway and down‐regulation of Cyt C and APAF‐1 protein expressions [37]. Guo et al. investigated the effect of the mitochondrial apoptosis pathway on global cerebral ischemia and demonstrated that GPR30 downregulates the P53‐PUMA signaling pathway, thereby inhibiting mitochondrial apoptosis and improving cerebral ischemia [38]. Xu et al. explored QSWZZP's neuroprotective effect and mechanism on CI/RI through the blood–brain barrier (BBB) and metabolomics. Their findings revealed that QSWZZP could reduce CI/RI‐induced BBB destruction by decreasing MMP‐9 and increasing claudin‐5 and occludin‐5 and further protected the brain, neurons and glial cells by regulating the lipid, fatty acid, and energy metabolisms [18].

P53 is an external AIF of mitochondrial apoptosis and primarily induces apoptosis via two pathways. First, it induces apoptosis by directly up‐regulating pro‐apoptotic proteins, such as B‐cell lymphoma protein 2 (BCL‐2)‐associated X (BAX) and PUMA, and downregulating anti‐apoptotic proteins, such as BCL‐2 and B‐cell lymphoma‐extra‐large (BCL‐XL). Second, the stimulated P53 induces mitochondrial stress response and subsequent formation of anti‐apoptotic protein BCL‐2 and BCL‐XL complexes, resulting in the opening of the mitochondrial membrane permeability transition pore and the entry of pro‐apoptotic proteins, such as Cyt C and AIF, into the cytoplasm. Deoxyadenosine triphosphate (dATP) subsequently prompts the binding of Cyt C to APAF‐1, which activates the caspase cascade reaction and triggers apoptosis [39]. N‐myc downstream‐regulated gene family member 4 (NDRG4) reduces CI/RI by inhibiting P53 expression and lowering the rate of neuronal apoptosis. These mechanisms include reducing the volume of cerebral infarction and decreasing the protein expression of P53, BAX, Cyt C, and caspase‐3 [40].

In the present study, we used UPLC‐Q‐TOF‐MS and metabolomics for the characterization and metabolite analysis of the chemical components of QSWZZP ex vivo and in vivo, while focusing on the P53/Cyt C/APAF‐1 mediated mitochondrial apoptosis pathway. Subsequently, we used molecular docking techniques and computer simulations to explore the effective target proteins and active ingredients of QSWZZP for cerebral ischemia treatment [41] and to validate the possible mitochondrial apoptosis pathway by which QSWZZP treats cerebral ischemia.

## 2 Materials and Methods

### 2.1 Drugs

QSWZZP (Batch No: 18099A, 210,400,109, NMPN: Z54020062) and nimodipine tablets (Batch No: BJ45200, NMPN: H20003010) were purchased from Xizang Ganlu Tibetan Medicine Co. Ltd. and Bayer Healthcare Co. Ltd., respectively.

### 2.2 Main Reagents and Antibodies

Ellagic acid (Batch No: PS010827), gallic acid (Batch No: PS000688), chlorogenic acid (Batch No: PS0131‐0025), caffeic acid (Batch No: PS010522), corilagin (Batch No: PS010034), quercetin (Batch No: MUST‐15090717), agarotetrol (Batch No: PS160823‐01), liquiritin (Batch No: PS141031‐01), luteolin (Batch No: PS000708), hesperidin (Batch No: C‐006‐170,216), crocetin 1(Batch No: PS000948), crocetin 11 (Batch No: PS000950), luteolin (Batch No: PS001232), and piperine (Batch No: PS08090901) were purchased from Chengdu Pusi Biotechnology Co. Ltd. The purity was > 99%, as determined using HPLC.

Cyt C (1:2000, GB11080), β‐actin rabbit monoclonal (1:1000, GB15003), and horseradish peroxidase‐labeled goat anti‐rabbit immunoglobulin G (1:10,000, CR2202048) antibodies and hematoxylin dye solution (CR22002071) were purchased from Servicebio. AIF (1:4000, 17,984‐1‐AP), P53 (1:10,000, 10,442‐1‐AP), and BCL‐XL (1:1000, 26,967‐1‐AP) rabbit polyclonal antibodies were purchased from Proteintech, and NDRG4 (1:1000, DF4341) and APAF‐1 (1:2000, AF0117) rabbit polyclonal antibodies were purchased from Affinity Biosciences.

### 2.3 Instruments and Equipment

UPLC (ACQUITY; Waters, USA) high resolution MS (Q‐TOF; Waters, USA) embedding machine (BMJ‐A; Changzhou Zhongwei Electronic Instrument Co. Ltd) pathological tissue bleaching oven (PHY‐III; Changzhou Zhongwei Electronic Instrument Co. Ltd.) automated stainer (RS36; Changzhou Paisijie Medical Equipment Co. Ltd) digital slice scanner (Panoramic 250; 3DHISTECH, Hungary) real‐time fluorescence quantification instrument (QuantStudio TM3; Thermo Fisher Scientific, USA) thermal cycler (TCA0096; Thermo Fisher Scientific, USA) electrophoresis system (PowerPac Basic; Bio‐Rad); and film‐based chemiluminescence imager (E‐BLOT XLI; Target Technology (Beijing) Co. Ltd).

### 2.4 Animals

Sprague–Dawley (SD) rats (specific pathogen free, male, 200–260 g, *n* = 235) were purchased from Chengdu Dashuo Experimental Animal Co. Ltd. (the experimental animal production license number: SCXK (Sichuan) 2020‐030, and the experimental animal quality certificate numbers: 51203500016830, 51203500017573, and 51203500031553). Most middle cerebral artery occlusion (MCAO) models, including those in the present study, have used male animals [42, 43, 44]. The reasons for this may be multifaceted. The effect of physiological fluctuations in estrogen levels on rat emotions precludes the advocation of using mixed‐sex animals for MCAO models [18, 45, 46]. Furthermore, several studies have reported the protective effect of estrogen on brain injury [47, 48, 49, 50]. Notably, the incidence of cerebral ischemia is significantly higher in men than in women.

The experimental protocol was approved by the Animal Ethics Committee of Chengdu University of Traditional Chinese Medicine (the experimental animal license number: SYXK (Sichuan) 2020‐124, and the experimental animal welfare ethics number: 2020‐39. date of approval: 22 May 2023).

### 2.5 UPLC‐Q‐TOF‐MS Test Conditions and Sample Preparation

#### 2.5.1 Chromatographic Conditions

Chromatographic separations were achieved using the ACQUITY UPLC BEH C_18_ column (2.1 mm × 150 mm, 1.7 μm). The mobile phase comprised an aqueous solution of 0.1% formic acid (A) and acetonitrile solution (B), with the flow gradient of eluent B at 95%–20% in 0–20 min and 20%–5% in 20–30 min. The column temperature was 30°C, the flow rate was 0.4 mL/min, and the injection volume was 2 μL.

#### 2.5.2 MS Conditions

The electrospray ionization (ESI) source was detected in positive and negative ion modes. The nitrogen flow rate was 600 L·h^−1^, the desolvation temperature was 350°C, the capillary voltage was 3.0 kV, the taper hole voltage was 30 kV, the collision energy was 15–45 eV, the ion source temperature was 120°C, and the scanning range was *m/z* 50–1500.

#### 2.5.3 Preparation of Test and Reference Solutions

QSWZZP powder (0.1000 g) was added to 10 mL of chromatography grade methanol and mixed thoroughly. The mixture was filtered through a 0.22 μm microporous filter [51] to obtain the test solution. The following reference compounds were precision weighed: tannic acid (4.68 μg), gallic acid (5.35 μg), chlorogenic acid (4.87 μg), caffeic acid (3.42 μg), corilagin (6.18 μg), quercetin (3.78 μg), agarotetrol (4.15 μg), glycyrrhizin (5.42 μg), luteoloside (6.20 μg), hesperidin (4.24 μg), saffron I (4.50 μg), saffron II (4.48 μg), luteolin (5.51 μg), and piperine (3.26 μg). Subsequently, they were each dissolved in 10 mL of methanol. Next, 1 mL of each reference solute was aspirated and mixed in 20 mL volumetric flasks, and added to 2 mL of the test solution to obtain the reference solution.

#### 2.5.4 Preparation of Serum, Tissue, and Plasma Samples

Male SD rats (*n* = 15) were divided into the sham‐operated (*n* = 3) and QSWZZP (*n* = 12) groups. The QSWZZP group was gavaged with a maximum dose of the QSWZZP solution (10 g/kg, 600‐fold the clinical adult dose of 0.0167 g/kg/day), and the sham‐operated group was gavaged an equal volume of physiological saline. Blood was collected from the abdominal aorta 30, 60, 90, and 120 min after drug administration and after the sham operation in the respective groups. The serum was separated, and 50 μL of the drug‐containing serum was aspirated at the four time points. Next, acetonitrile precipitant (1000 μL) was added, and the solution was vortexed for 2 min and centrifuged for 10 min (13,000 r/min, 4°C). The supernatant (2 mL) was dried with a nitrogen blower (37°C), dissolved in 200 μL of methanol, mixed well, centrifuged, and filtered. After blood collection, the brain, liver, kidney, and urine of each animal were resected, and 0.1000 g of the brain, liver, and kidney samples were precision weighed for homogenization. The aforementioned processing method was used. The urine sample was prepared similar to the serum samples.

Male SD rats (*n* = 40) were divided into a sham‐operated, model control, nimodipine [52] control, and QSWZZP groups, with 10 rats in each group. The QSWZZP group was gavaged the optimal dose of the QSWZZP solution (66.68 mg/kg, equivalent to four‐fold the clinical adult dose of 0.0167 g/kg/day) [53, 54]. The nimodipine control group was gavaged an equivalent dose of nimodipine, and the sham‐operated and model control groups were gavaged an equal volume of physiological saline. After anticoagulation treatment, whole blood was collected from each group and centrifuged at 3500 r/min and 4°C for 10 min to separate and collect the plasma. Subsequently, 200 μL plasma per animal was precisely aspirated and placed in a 2.0 mL Eppi tube and processed similarly to the serum samples.

#### 2.5.5 Data Processing and Analysis

UPLC‐Q‐TOF‐MS was used to detect and analyze the aforementioned samples. MS data were analyzed using the MassLynx V4.2 software. The control substance was referenced, and the chemical composition database of QSWZZP was used to preliminarily identify the QSWZZP chemical components, as well as their distribution in the blood and tissues. The ion information of the MS fragments was used to infer fragmentation patterns, and the corresponding mass‐to‐charge ratio (*m/z*) and ion peak intensity from the Human Metabolome Database (HMDB; https://hmdb.ca/spectra/ms‐ms/search) were used for qualitative analysis to obtain metabolite information. Semi‐quantitative and normalization processing was conducted using the peak area of each peak and used as the final data for statistical analysis.

### 2.6 Animal Grouping and Dose Design

Male SD rats (*n* = 180) were randomized into three batches, with six groups in each batch, and 10 animals in each group: sham‐operated, model control, nimodipine control (30 mg/kg/day, equivalent to five‐fold the daily clinical adult dose), QSWZZP low‐dose (33.34 mg/kg/day, two‐fold the daily clinical adult dose), QSWZZP medium‐dose (66.68 mg/kg/d, four‐fold the daily clinical adult dose, optimal dose), and QSWZZP high‐dose (133.36 mg/kg/d, eight‐fold the daily clinical adult dose) groups. Experimental animals are randomized via a computer‐generated random number table: each animal receives a unique identifier, is stratified by variables (body weight) to reduce bias, is assigned a corresponding random number, and is allocated to groups based on sorted random numbers. This process is conducted by an independent researcher not involved in subsequent data collection/analysis to ensure objectivity and reproducibility.

The MCAO model prophylactic (10 mL/kg/day, once daily) was gavaged to each group for 7 consecutive days. The sham‐operated and model control groups were gavaged corresponding volumes of physiological saline. The first batch was used for neurobehavioral evaluation and calculation of cerebral infarction ratio. The second batch was used for brain tissue histopathology (hematoxylin and eosin (HE) staining), positive expression of apoptotic cells in brain tissue (terminal deoxynucleotidyl transferase‐mediated deoxyuridine triphosphate nick end labelling (TUNEL) staining) and Cyt C in the brain interstitial region and immunofluorescence (IF) detection of APAF‐1 and NDRG4 expression. The third batch was used for mRNA expression (Reverse transcription polymerase chain reaction, RT‐PCR) and protein expression (western blot, WB) detection of mitochondrial apoptosis pathway‐related factors in brain tissue.

### 2.7 Preparation of the MCAO Model

The MCAO model was prepared using the thread occlusion method. After the rats were anesthetized with an intraperitoneal injection of 2% sodium pentobarbital (45 mg/kg body weight), the mid‐line of the neck was incised, and the right common carotid artery (CCA) was blunt dissected. The bifurcation of the internal carotid artery (ICA) and external carotid artery (ECA) was identified along the CCA, and the ECA was ligated. The pterygopalatine artery was identified along the ICA and ligated. Using a self‐made needle picking technique, a 0.26‐mm monofilament nylon thread was inserted into the ICA at a distance of approximately 20 mm (i.e., the mark of the suture line reached the bifurcation of the ICA and ECA) to block the MCA. The suture thread was fixed, the incision was sutured, and an appropriate amount of gentamicin solution was added for disinfection. At 2 h after cerebral ischemia, the nylon thread was gently removed to achieve reperfusion. The sham‐operated group underwent only suture preparation [18, 43].

### 2.8 Neurobehavioral Rating and Cerebral Infarction Ratio

The neurobehavioral rating of rats was estimated at 2 h and 24 h after cerebral ischemia using the Bederson (0–5) score [55]. The grades are as follows: ‘−’, no symptoms of nerve injury. ‘+’, the left forelimb not fully extended. ‘++’, circling to the left. ‘+++’, slumping to the left, and ‘++++’, the rats display no movement and lose consciousness.

At 24 h after cerebral ischemia, the animals were anesthetized and euthanized before the brains were removed *in toto*. The brains were weighed, stored for 30 min at −20°C, divided into five equal pieces, stained with freshly prepared 1% triphenyl tetrazolium chloride buffer (30 min, 37°C, under dark conditions and flipped every 15 min). The brain tissues were fixed in 10% formaldehyde, and the infarcted area was separated, weighed, and the infarct ratio was calculated as follows: infarct ratio = weight of infarcted area (in grams)/weight of brain tissue (in grams) × 100%.

### 2.9 HE And TUNEL Staining

#### 2.9.1 HE Staining

The formalin‐fixed brain tissues were stained with HE and observed using the Pannoramic 250 digital slice scanner at 100× and 400× magnification. Neuronal cell necrosis in the cortex, hippocampal CA1 and CA3 regions, and diencephalon were assessed as follows: no lesion (−), mild (+), mild (++), moderate (+++), and severe (+++).

#### 2.9.2 TUNEL Staining

The formalin‐fixed brain tissues were subjected to TUNEL staining at different solution ratios. For apoptotic cell analysis, the Pannoramic 250 digital slice scanner was used to obtain images at 400× magnification of three fields (three images per field). The rate of apoptosis‐positive cells in the image (%) was calculated.

### 2.10 Molecular Docking

#### 2.10.1 Screening and Acquisition of Potential Active Ingredients and Target Proteins

Ninety‐three chemical components (75 formulation, 15 blood‐entry, and three brain‐entry components) that were identified from previous studies (*n* = 42) and the present study (*n* = 33) were analyzed using the SwissADME platform (http://www.swissadme.ch/). The platform [56, 57, 58] screens for potential active ingredients, with a screening criteria of at least two ‘yes’ outcomes for the following items: gastrointestinal absorption, high, and drug likeness. The PubChem small molecule compound structure library (https://pubchem.ncbi.nlm.nih.gov/) was used to screen the three‐dimensional sdf file of the potential active ingredients. If a component's structure was not found in PubChem, Chem3D 20.0 was used to draw and save its structural formula in sdf format. The pdb file of AIF (PDB ID: 5KVI), P53 (PDB ID: 2J1X), Cyt C (PDB ID: 5TY3), APAF‐1 (PDB ID: 1Z6T), and NDRG4 (PDB ID: AF‐Q9ULP0‐F1) were searched and downloaded from the PDB database (https://www.rcsb.org/).

#### 2.10.2 Preprocessing of Potential Active Ingredients and Target Proteins

The Open Babel GUI software was used to convert the potential active ingredient sdf files into pdb files. The pdbqt files were subsequently exported using the AutoDockTools‐1.5.7 software. The target protein was processed by first removing water and solvent molecules using the Pymol 2.1 software and subsequently adding full hydrogen using the AutoDockTools‐1.5.7 software. The file was finally saved as a pdbqt file [59].

#### 2.10.3 Molecular Docking of Potential Active Ingredients and Target Proteins

The AutoDockTools‐1.5.7 software was used to dock the obtained potential active ingredients (pdbqt files) and five target proteins (pdbqt files) individually. The protein molecules were set to rigid docking, with a docking frequency of 50 times. The obtained docking results were visualized using Pymol 2.1 [59, 60, 61].

### 2.11 RT‐PCR and WB

#### 2.11.1 RT‐PCR

TRIzol‐homogenized ischemic brain tissue samples were treated with 0.2 mL of chloroform, 1 mL of 75% ethanol, and 10 μL of LDEPC. Next, 5 × gDNA Eraser Buffer (2 μL), gDNA Eraser (1 μL), RNA (2 μL), and RNase Free dH_2_O (5 μL) were reacted at 42°C for 2 min. Subsequently, 5 × PrimeScript Buffer (24 μL), PrimeScript RT Enzyme Mix I (1 μL), RT Primer Mix (1 μL), and RNase Free dH_2_O (4 μL) were made to react in a PCR instrument. Subsequently, 2 × Real PCR EasyTM Mix SYBR (10 μL), 10 μm forward primer (0.8 μL), and 10 μM reverse primer (0.8 μL) were added. The RT‐PCR was performed with the template cDNA (2 μL) and dH_2_O (6.4 μL). The relative AIF of X was calculated using the 2^−△△CT^ method. The APAF‐1, BCL‐XL, cleaved caspase 8, Cyt C, and P53 mRNA expression levels (expressed as differential multiples of XmRNA expression in 2^−△△*CT*^) were calculated as follows:

$$△{\mathrm{CT}}_{\text{Intervention}}={\mathrm{CT}}_{\text{Objective of intervention}}-{\mathrm{CT}}_{\text{Intervention internal reference}}$$

$$△{\mathrm{CT}}_{\text{Blank}}={\mathrm{CT}}_{\text{Blank purpose}}-{\mathrm{CT}}_{\text{Blank internal reference}}$$

$$△△\mathrm{CT}=△{\mathrm{CT}}_{\text{Intervention}}-△{\mathrm{CT}}_{\text{Blank}}$$

#### 2.11.2 Western Blot

Brain tissue samples (20 mg) were ground in a solution comprising lysate, protease inhibitor, and phenylmethylsulfonyl fluoride (100:1:1). The resulting solution was centrifuged, and the supernatant was separated to obtain the total protein. A protein standard solution (1600 g/mL) was diluted into 1600, 800, 400, 200, 100, 50, 25, and 0 g/mL series sequentially. After 30 min (at 37°C), the optical density (OD) at 562 nm was measured, and the OD and concentration standard curves were generated. Each total protein supernatant sample (1 L) was diluted 50‐fold in 49 L of lysis solution, and 20 L was added to 96‐well plates. Next, 200 L of BCA working solution, comprising BCA reagents A and B (50:1), was mixed well and incubated for 30 min at 37°C. The OD of the sample at 562 nm was measured, and the protein content was calculated according to the standard curve. Sample electrophoresis, membrane transfer, closure, antibody incubation, development, fixation, and image analysis were performed after protein denaturation, and the results were presented as the relative target protein expression levels (integrated OD of the target protein/internal integrated OD of the internal reference).

### 2.12 Immunofluorescence

Three indicators, Cyt C, APAF‐1, and NDRG4, were selected for IF testing based on the WB results of the sham‐operated, model control, nimodipine control, and QSWZZP medium‐dose groups. The formalin‐fixed brain tissue was dehydrated, embedded, sliced, and dewaxed. After antigen retrieval, goat serum blocking solution was added dropwise, and blocking was performed at 25°C for 20 min. Subsequently, the primary antibody was added, incubated overnight at 4°C, and washed with phosphate‐buffered saline (PBS) thrice (5 min/wash). Next, the secondary antibody was added and incubated for 30 min at 37°C, and washed with PBS thrice (5 min/wash). The reaction was sealed using an anti‐fluorescence attenuation sealing agent. A microscope camera system was used to capture three images at 200× magnification, and the fluorescence intensity and area were measured using the Image‐J software. The average fluorescence intensity of each image was calculated. Cell nuclei were stained blue with 4′,6‐diamidino‐2‐phenylindole staining, whereas Cyt C, APAF‐1, and NDRG4 fluorescence were stained green.

### 2.13 Statistical Analysis

Plasma metabolites of each group were analyzed using the SIMCA 14.1 software. Principal component analysis (PCA) and orthogonal partial least squares discriminant analysis (OPLS‐DA) were performed on the plasma metabolites in each group using normalized values.

Experimental data were represented as mean ± standard deviation. The SPSS 19.0 for Windows software was used for all statistical analyses. Homogeneity of variance was conducted using the Levene test. *p* > 0.05 indicated homogeneity of variance, and a t‐test or one‐way analysis of variance (ANOVA) was used to conduct a significance test for mean differences between two or more groups of data in the independent samples. *p* < 0.05 indicated uneven variance, and the ordinal data were compared for inter‐group differences using the non‐parametric Mann–Whitney test. Furthermore, the continuous data were compared for inter‐group differences using the non‐parametric Mann–Whitney test.

## 3 Results

### 3.1 Chemical Characterization of QSWZZP

**FIGURE 1:**
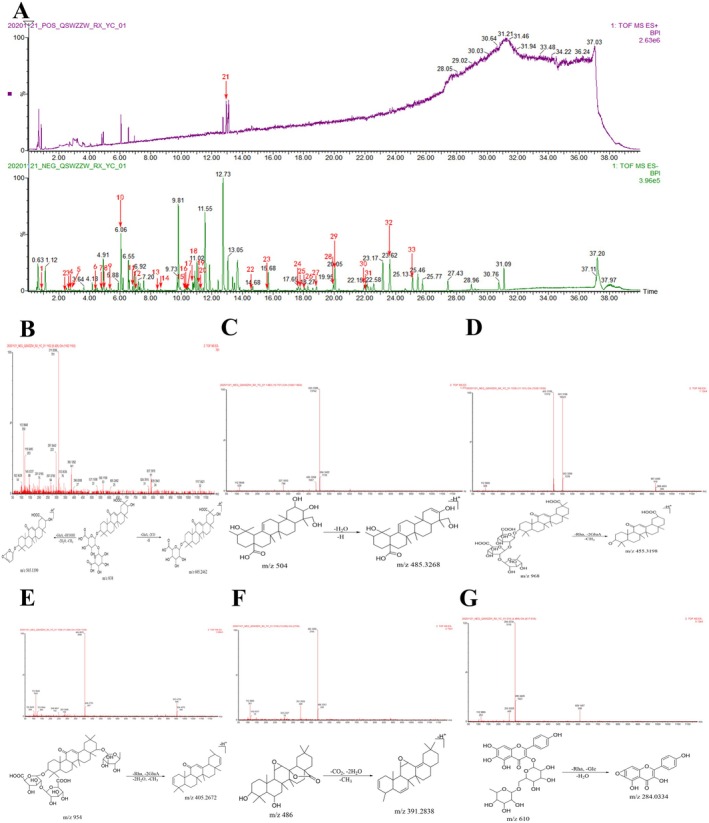
Chemical composition of QSWZZP and their mass spectra and cleavage patterns (A) Positive and negative base‐peak ion flow (BPI) diagrams. (B) Macedonoside A. (C) 2α, 3β, 19α, 23‐tetrahydroxyurs‐12‐en‐28‐oic acid. (D) Rhaglycyrrhizin. (E) Uralicus glycyrrhizae saponin T. (F) 3β, 6β‐dihydroxy‐11α, 12α‐epoxyoleanol‐13β, 28‐lactone. (G) 6‐Hydroxykaempferol‐3‐O‐beta‐rutinoside.

The positive and negative base‐peak ion (BPI) flow diagrams are presented in Figure 1A. Based on the 42 previously identified QSWZZP chemical constituents [9, 30] and the retention time of the positive and negative BPI current chromatogram and the information of the first and second fragment ions detected via UPLC‐Q‐TOF‐MS, 33 chemical constituents (one in positive ion mode and 32 in negative ion mode) were analyzed and identified. These included 13 triterpenoids (e.g., Macedonoside A, Macedonoside E, etc.), nine flavonoids (e.g., hydroxy saffron yellow pigment A, (8R)‐Evofolin B, etc.), and two anthraquinones (rhododendron methyl ether‐8‐O‐β‐D‐gentian disaccharide and 1,7‐dimethoxy‐2,8‐dihydroxy‐3‐methylanthraquinone‐2‐O‐β‐D‐glucoside). Two chromones (butyroside I and 6‐methoxy‐2‐(2‐(3‐methoxyphenyl) ethyl) chromone) and seven other constituents (3‐{[6‐O‐(D‐Galactopyranosyl)‐β‐D‐galactopyranosyl] oxy}‐1,2‐propan‐ediyl diacetate, acetovanillone, etc) were identified as well (Table 1).

**TABLE 1:** Information on the identification of chemical constituents in QSWZZP.

Serial number	*t*_R_ (min)	English name	Molecular formula	Ion mode	Fragmentation information (m/z)			Categorization	Possible sources
					Parent ion		Daughter ion		
					Measured value	Theoretical value			
1	0.85	3‐{[6‐O‐(D‐Galactopyranosyl)‐β‐D‐galactopyranosyl] oxy}‐1,2‐propan‐ediyl diacetate	C_19_H_32_O_15_	[M‐H]^−^	499.1707	499.1663	—	Other categories	*Carthamus tinctorius* L.
2	2.49	Apocynin	C_9_H_10_O_3_	[M‐H]^−^	165.0589	165.0552	119.0534	Other categories	*Styrax benzoin* Dryand.
3	2.64	Biflorin	C_16_H_18_O_9_	[M‐H]^−^	353.0843	353.0873	233.0468, 205.0534	Chromones	*Syzygium aromaticum* (L.) Merr. & L.M.Perry.
4	2.71	2‐Methoxy‐1, 4‐biphenol‐1‐O‐[6‐O‐(3‐methoxy‐4‐hydroxybenzoyl)] ‐β‐D‐glucopyranoside	C_21_H_24_O_11_	[M‐H]^−^	451.1240	451.1240	373.0800, 351.0768	Other categories	*Amomum acre* Valeton.
5	2.76	Hydroxysafflor yellow A	C_27_H_32_O_16_	[M‐H]^−^	611.1655	611.1612	491.1202, 403.1092, 325.0745	Flavonoids	*Carthamus tinctorius* L.
6	4.49	6‐Hydroxykaempferol 3‐O‐β‐rutinoside	C_27_H_30_O_16_	[M‐H]^−^	609.1487	609.1456	284.0334	Flavonoids	*Carthamus tinctorius* L.
7	4.79	(8R)‐Evofolin B	C_17_H_18_O_6_	[M‐H]^−^	317.1034	317.1025	363.1072	Flavonoids	*Agastache rugosa* (Fisch. & C.A.Mey.) Kuntze., *Cinnamomum cassia* (L.) J.Presl
8	4.93	Isoliquirtin apiside	C_26_H_30_O_13_	[M‐H]^−^	549.1614	549.1608	255.0666, 135.0072, 119.0495	Flavonoids	*Glycyrrhiza uralensis* Fisch.
9	5.29	3’‐Hydroxy‐8‐methoxyvestitol	C_17_H_18_O_6_	[M‐H]^−^	317.1034	317.1025	363.1072, 299.0979	Flavonoids	*Dalbergia odorifera* T.C.Chen.
10	6.07	Physcion‐8‐O‐β‐D‐gentiobioside	C_28_H_32_O_15_	[M‐H]^−^	607.1698	607.1663	284.0334	Anthraquinones	*Senna tora* (L.) Roxb.
11	6.84	Neoliquiritin	C_21_H_22_O_9_	[M‐H]^−^	417.1207	417.1186	255.0666, 135.0072, 119.0495	Flavonoids	*Glycyrrhiza uralensis* Fisch.
12	7.08	1,7‐Dimethoyl‐2,8‐dihydroxyl‐3‐methlanthraquinone‐2‐O‐β‐D‐glucopyranoside	C_23_H_24_O_11_	[M‐H]^−^	475.1235	475.1240	283.1740, 267.0642, 135.0112, 119.0495	Anthraquinones	*Senna tora* (L.) Roxb.
13	8.43	Macedonoside A	C_42_H_62_O_17_	[M‐H]^−^	837.3970	837.3909	605.2462, 565.1190	Triterpenes	*Glycyrrhiza uralensis* Fisch.
14	8.65	Safflospermidine A/Safflospermidine B	C_34_H_37_N_3_O_6_	[M‐H]^−^	582.2558	582.2604	462.2020, 342.1463, 119.0495	Other categories	*Carthamus tinctorius* L.
15	10.22	4′, 7‐Dimethoxyisoflavone	C_17_H_14_O_4_	[M‐H]^−^	281.0814	281.0814	—	Flavonoids	*Glycyrrhiza uralensis* Fisch.
16	10.26	Macedonoside E	C_42_H_62_O_17_	[M‐H]^−^	837.3970	837.3909	823.4117, 485.3423, 351.0570	Triterpenes	*Glycyrrhiza uralensis* Fisch., *Fel* Ursi
17	10.34	Formononetin	C_16_H_12_O_4_	[M‐H]^−^	267.0642	267.0657	—	Flavonoids	*Dalbergia odorifera* T.C.Chen., *Glycyrrhiza uralensis* Fisch., *Syzygium aromaticum* (L.) Merr. & L.M.Perry
18	10.75	23‐Hydroxytormentic acid	C_30_H_48_O_6_	[M‐H]^−^	503.3389	503.3373	485.3268	Triterpenes	*Syzygium aromaticum* (L.) Merr. & L.M.Perry.
19	11.15	Rhaglycyrrhizin	C_48_H_72_O_20_	[M‐H]^−^	967.4586	967.4539	455.3198	Triterpenes	*Glycyrrhiza uralensis* Fisch.
20	11.29	Uralsaponin T	C_48_H_74_O_19_	[M‐H]^−^	953.4758	953.4746	405.2672	Triterpenes	*Glycyrrhiza uralensis* Fisch.
21	12.94	6‐Methoxy‐2‐[2‐(3‐methoxyphenyl) ethyl]chromone	C_19_H_18_O_4_	[M + H]^+^	311.1257	311.1283	205.0529, 177.0562	Chromones	*Aquilaria sinensis* (Lour.) Spreng.
22	14.58	Asiatic acid	C_30_H_48_O_5_	[M‐H]^−^	487.3448	487.3423	—	Triterpenes	*Syzygium aromaticum* (L.) Merr. & L.M.Perry.
23	15.64	3β, 6β‐Dihydroxy‐11α, 12α‐epoxyolean‐13β, 28‐olide	C_30_H_46_O_5_	[M‐H]^−^	485.3268	485.3267	391.2838	Triterpenes	*Styrax benzoin* Dryand.
24	17.63	Sumaresinolic acid	C_30_H_48_O_4_	[M‐H]^−^	471.3490	471.3474	—	Triterpenes	*Styrax benzoin* Dryand.
25	17.79	Maslinic acid	C_30_H_48_O_4_	[M‐H]^−^	471.3490	471.3474	—	Triterpenes	*Nepeta angustifolia* C.Y.Wu., *Terminalia bellirica* (Gaertn.) Roxb., *Syzygium aromaticum* (L.) Merr. & L.M.Perry, *Terminalia chebula* Retz.
26	18.03	Corosolic acid	C_30_H_48_O_4_	[M‐H]^−^	471.3490	471.3474	—	Triterpenes	*Nepeta angustifolia* C.Y.Wu., *Syzygium aromaticum* (L.) Merr. & L.M.Perry.
27	18.84	(9Z,11E)‐13‐Oxooctadeca‐9,11‐dienoic acid	C_18_H_30_O_3_	[M‐H]^−^	293.2110	293.2117	—	Other categories	*Carthamus tinctorius* L.
28	19.95	Hemerocallal A	C_20_H_28_O_2_	[M‐H]^−^	299.2014	299.2011	149.0967	Other categories	*Boswellia carteri* Birdw.
29	20.05	Siaresinolic acid	C_30_H_48_O_4_	[M‐H]^−^	471.3490	471.3474	—	Triterpenes	*Styrax benzoin* Dryand.
30	21.95	Ursolic acid	C_30_H_48_O_3_	[M‐H]^−^	455.3498	55.3525	—	Triterpenes	*Dalbergia odorifera* T.C.Chen, *Nepeta angustifolia* C.Y.Wu., *Lagotis brachystachya* Maxim., *Syzygium aromaticum* (L.) Merr. & L.M.Perry, *Carthamus tinctorius* L., *Terminalia bellirica* (Gaertn.) Roxb., *Phyllanthus emblica* L.
31	22.02	Oleanolic acid	C_30_H_48_O_3_	[M‐H]^−^	455.3498	455.3525	—	Triterpenes	*Styrax benzoin* Dryand., *Nepeta angustifolia* C.Y.Wu., *Sarcandra glabra* (Thunb.) Nakai., *Glycyrrhiza uralensis* Fisch., *Phyllanthus emblica* L., *Terminalia bellirica* (Gaertn.) Roxb., *Syzygium aromaticum* (L.) Merr. & L.M.Perry
32	23.62	Linoleic acid	C_18_H_32_O_2_	[M‐H]^−^	279.2317	279.2324	—	Other categories	*Terminalia chebula* Retz., *Codonopsis xizangensis* D.Y.Hong., *Zingiber officinale* Roscoe., *Nigella glandulifera* Freyn & Sint.
33	25.13	Glyasperin C	C_21_H_24_O_5_	[M‐H]^−^	355.1573	355.1545	323.2180, 255.2353	Flavonoids	*Glycyrrhiza uralensis* Fisch.

#### 3.1.1 Triterpenoids

Pentacyclic triterpenoids were primarily identified in QSWZZP. The most common skeleton was an oleanane skeleton (compounds 14, 17, 20, 21, 24, 25, 26, and 32) followed by an ursane skeleton (compounds 19, 23, 27, 30, and 31) [62]. Two glucuronides (2GluA) or two glucuronides and a rhamnose (2GluA + Rha) were attached at the C‐3 position, or rhamnose (Rha) was attached at the C‐22 position to form triterpene saponin analogues (compounds 14, 17, 20, and 21), which were mainly derived from *Glycyrrhiza uralensis*. The remaining compounds were obtained by changing the double bond position based on the pentacyclic triterpene nucleus and increasing ‐CH_3_, ‐OH, ‐COOH, H_2_O, or CO. The cleavage patterns of three triterpenoids are described herewith:

Macedonoside A (Compound 14): The UPLC retention time was 8.43 min. The compound exhibited a quasi‐molecular ion peak [M‐H]^−^ at *m/z* 837.3970, indicating a molecular weight of 838. A molecular formula of C_42_H_62_O_17_ was obtained after a comparative analysis with PubChem. Based on the secondary MS fragment ions (*m/z* 605.2462 [M‐GluA‐2CO‐H]^−^ and *m/z* 565.1190 [M‐GluA‐HCOOH‐2H_2_O‐CH_3_]^−^) and the available literature [63], the compound was identified as Macedonoside A. The secondary MS and cleavage patterns are illustrated in Figure 1B.

- 2α,3β,19α,23‐tetrahydroxyurs‐12‐en‐28‐oic acid (Compound 19): The UPLC retention time was 10.75 min. The compound exhibited a quasi‐molecular ion peak [M‐H]^−^ at *m/z* 503.3389, detected using primary MS against secondary MS, indicating a molecular weight of 504. A molecular formula of C_30_H_48_O_6_ was obtained after a comparative analysis with PubChem. Based on the secondary MS fragment ion (*m/z* 485.3268 [M‐H_2_O‐H]^−^) and available literature [64], the compound was identified as 2α,3β,19α,23‐tetrahydroxyurs‐12‐en‐28‐oic acid (23‐hydroxytormentic acid). The secondary MS and cleavage patterns are illustrated in Figure 1C.
- Rhaglycyrrhizin (Compound 20). The UPLC retention time was 11.15 min. The compound exhibited a quasi‐molecular ion peak [M‐H]^−^ at *m/z* 967.4586, indicating a molecular weight of 968. A molecular formula of C_48_H_72_O_20_ was obtained after a comparative analysis with PubChem. Based on the characteristic secondary MS fragment ion *m/z* 455.3198 [M‐Rha‐2GluA‐CH3]^−^ and available literature, the compound was identified as rhaglycyrrhizin [65]. The secondary MS and cleavage patterns are illustrated in Figure 1D.

#### 3.1.2 Flavonoids

**FIGURE 2:**
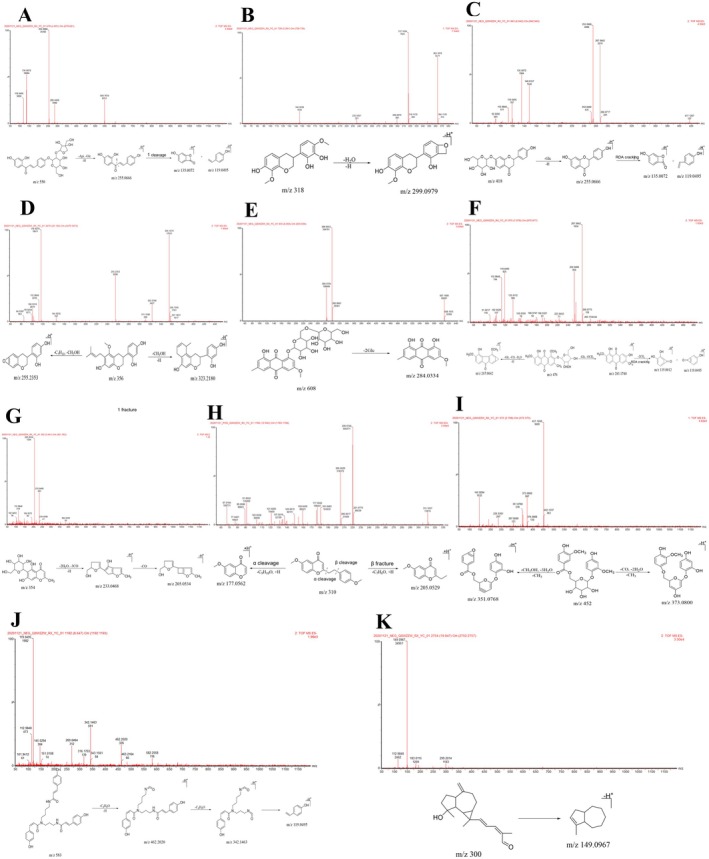
Chemical composition of QSWZZP and its mass spectra and cleavage patterns (A) Isoliquiritin apioside. (B) 3′‐Hydroxy‐8‐methoxyvestitol. (C) Neoliquiritin. (D) Glyasperin C. (E) Physcion‐8‐O‐β‐gentiobioside. (F) 1,7‐dimethoxy‐2,8‐dihydroxy‐3‐methylanthraquinone‐2‐O‐β‐D‐glucoside. (G) Syrigin. (H) 6‐methoxy‐2‐[2‐(3′‐methoxyphenyl) ethyl] chromone (I) 2‐methoxy‐1,4‐diphenol‐1‐O‐[6‐O‐(3‐methoxy‐4‐hydroxybenzoyl)]‐β‐D‐glucopyranoside). (J) Safflospermidine A/safflospermidine B. (K) Hemerocallal A.

Nine flavonoids were newly identified in this study. The flavonoid nucleus was often combined with a five‐carbon sugar (apiose, 132 Da), six‐carbon sugar (glucose, 162 Da), and six‐carbon uronic acid (glucuronic acid, 176 Da) units to form flavonoid glycosides. Hence, the sugar group fragments often appeared when cracking. Moreover, the flavonoid parent nucleus was susceptible to Retro Diels‐Alder (RDA) cleavage, resulting in the formation of characteristic fragment ions at *m/z* 135.0072 and 119.0495 [66, 67]. Additionally, the flavonoid constituents had certain commonly lost fragment ions, such as ^−^H_2_O (18 Da), ^−^CO (28 Da), ^−^CH_2_OH (31 Da), ^−^C_8_H_8_O (120 Da), and ^−^C_5_H_10_ (70 Da). The cleavage patterns of three flavonoids are described herewith:

- 6‐Hydroxy kaempferol‐3‐O‐β‐rutinoside (Compound 6): The UPLC retention time was 4.49 min. The compound exhibited a quasi‐molecular ion peak [M‐H]^−^ at *m/z* 609.1487, indicating a molecular weight of 610. A molecular formula of C_27_H_30_O_16_ was obtained after a comparative analysis with PubChem. Based on the characteristic secondary MS fragment ion (*m/z* 284.0334) and available literature [68], the compound was identified as 6‐hydroxykaempferol 3‐O‐β‐rutinoside (6‐hydroxykaempferol 3‐O‐β‐rutinoside). The structural formula and secondary MS and cleavage patterns are illustrated in Figure 1F.
- Apigenin isoglycoside (Compound 8): The UPLC retention time was 4.93 min. The compound exhibited a quasi‐molecular ion peak [M‐H]^−^ at *m/z* 549.1614, detected using primary MS against secondary MS, indicating a molecular weight of 550. A molecular formula of C_26_H_30_O_13_ was obtained after a comparative analysis with PubChem. Based on the molecular ion peak (*m/z* 255.0666 [M‐Api‐Glc‐H]‐), characteristic secondary MS fragment ions (*m/z* 135.0072 and 119.0495), and available literature [69], the compound was identified as isoliquiritin apioside. The secondary MS and cleavage patterns are illustrated in Figure 2A.
- 3’‐Hydroxy‐8‐methoxyvestitol (Compound 9): The UPLC retention time was 5.29 min The compound exhibited a quasi‐molecular ion peak [M‐H]^−^ at *m/z* 317.1034, indicating a molecular weight of 318. A molecular formula of C_17_H_18_O_6_ was obtained after a comparative analysis with PubChem. Based on the molecular peak ion (*m/z* 363.1072 [M + HCOOH‐H]^−^), primary MS fragment ion (*m/z* 299.0979 [M‐H_2_O‐H]^−^), and available literature [70], the compound was identified as 3′‐hydroxy‐8‐methoxyvestitol. The primary MS and cleavage patterns are illustrated in Figure 2B.

#### 3.1.3 Anthraquinones

Two anthraquinones (emodin methyl ether‐8‐O‐β‐D‐gentiobioside and 1,7‐dimethoxy‐2,8‐dihydroxy‐3‐methylanthraquinone‐2‐O‐β‐D‐glucoside) were identified. The cleavage characteristics were similar to those of the flavonoids. The anthraquinone glycosides could be deglycosylated to form anthraquinone mother nuclei, which were susceptible to de^−^CO, ^−^H_2_O, and ^−^OCH_3_ with the hydroxyl groups attached to them and to RDA cleavage to form characteristic ions at *m/z* 135.0072 and 119.0495. The cleavage patterns are described herewith:

- Emodin methyl ether‐8‐O‐β‐D‐gentiobioside (Compound 10): The UPLC retention time was 6.07 min. The compound exhibited a quasi‐molecular ion peak [M‐H]^−^ at *m/z* 607.1698, indicating a molecular weight of 608. A molecular formula of C_28_H_32_O_15_ was obtained after a comparative analysis with PubChem. Based on the secondary MS fragment ion (*m/z* 284.0334) and available literature [71], the compound was identified as physcion‐8‐O‐β‐D‐gentiobioside. The secondary MS and cleavage patterns are illustrated in Figure 2E.
- 1,7‐dimethoxy‐2,8‐dihydroxy‐3‐methylanthraquinone‐2‐O‐β‐D‐glucoside (Compound 12): The UPLC retention time was 7.08 min. The compound exhibited a quasi‐molecular ion peak [M‐H]^−^ at *m/z* 475.1235, indicating a molecular weight of 476. A molecular formula of C_23_H_24_O_11_ was obtained after a comparative analysis with PubChem. Based on the secondary MS fragment ions (*m/z* 283.1740 [M‐Glc‐OCH3]^−^, 267.0642 [M‐Glc‐CO‐H_2_O‐H]^−^, 135.0072, and 119.0495) and available literature [72], the compound was identified as 1,7‐dimethoxy‐2,8‐dihydroxy‐3‐methylanthraquinone‐2‐O‐β‐D‐glucopyranoside (1,7‐dimethoyl‐2,8‐dihydroxyl‐3‐methlanthraquinone‐2‐O‐β‐D‐glucopyranoside). The secondary MS and cleavage patterns are illustrated in Figure 2F.

#### 3.1.4 Chromones

Owing to the different structural formulas, the cracking rules of chromone compounds are inconsistent as well. Among them, the easily lost fragments are^−^CO and^−^H_2_O. The identified chromone components were syringin I (from *Syzygium aromaticum* ) and 6‐methoxy‐2‐(2‐(3‐methoxyphenyl) ethyl) chromone (from *Aquilaria sinensis* ). The cleavage patterns are described herewith:

- Syringin I (Compound 3): The UPLC retention time was 2.64 min. The compound exhibited a quasi‐molecular ion peak [M‐H]^−^ at *m/z* 353.0843, indicating a molecular weight of 354. A molecular formula of C_16_H_18_O_9_ was obtained after a comparative analysis with PubChem. Based on the secondary MS fragment ions (*m/z* 233.0468 [M‐2H_2_O‐3CO‐H]^−^ and 205.0534 [M‐2H_2_O‐4CO‐H]^−^) and available literature [64], the compound was identified as butyroside I (biflorin). The secondary MS and cleavage patterns are illustrated in Figure 2G.
- 6‐methoxy‐2‐(2‐(3‐methoxyphenyl) ethyl) chromone (Compound 21): The UPLC retention time was 12.94 min. The compound exhibited an excimer ion peak [M + H]^+^ at *m/z* 311.1257, indicating a molecular weight of 310. A molecular formula of C_19_H_18_O_4_ was obtained after a comparative analysis with PubChem. Based on the secondary MS fragment ions (*m/z* 205.0529) and ion peaks (*m/z* 177.0562) and available literature [73], the compound was identified as 6‐methoxy‐2‐(2‐(3‐methoxyphenyl) ethyl) chromone (6‐methoxy‐2‐[2‐(3‐methoxyphenyl) ethyl] chromone). The secondary MS and cleavage patterns are illustrated in Figure 2H.

#### 3.1.5 Other Compound Classes

Other compound classes included phenols, organic acids, steroids, and phenolamines among others. The phenols were vanilla ethanone and 2‐methoxy‐1,4‐diphenol‐1‐O‐[6‐O‐(3‐methoxy‐4‐hydroxybenzoyl)]‐β‐D‐glucopyranoside. Organic acids were mainly fatty acids ((9Z,11E)‐13‐oxooctadeca‐9,11‐dienoic acid and linoleic acid), steroids included hemerocallal A, and phenolic amines included safflospermidine A/B. In addition, an unknown compound was found, 3‐{[6‐O‐(D‐galactopyranosyl)‐β‐D‐galactopyranosyl] oxy}‐1,2‐propanediyl diacetate. The compounds were mainly derived from *Carthamus tinctorius* L., *Amomum acre* Valeton, *Styrax benzoin* Dryand, *Terminalia chebula* Retz, *Codonopsis xizangensis* D. Y. Hong, *Zingiber officinale* Roscoe, *Nigella glandulifera* Freyn & Sint. The cleavage patterns of the three compounds are described herewith.

- 2‐methoxy‐1,4‐diphenol‐1‐O‐[6‐O‐(3‐methoxy‐4‐hydroxybenzoyl)]‐β‐D‐glucopyranoside (Compound 4): The UPLC retention time was 2.71 min. The compound exhibited a quasi‐molecular ion peak [M‐H]^−^ at *m/z* 451.1240, indicating a molecular weight of 452. A molecular formula of C_21_H_24_O_11_ was obtained after a comparative analysis with PubChem. Based on the primary MS fragment ions (*m/z* 373.0800 and 351.0768) and available literature [74], the compound was identified as 2‐methoxy‐1,4‐diphenol‐1‐O‐[6‐O‐(3‐methoxy‐4‐hydroxybenzoyl)]‐β‐D‐glucopyranoside (2‐methoxy‐1,4‐biphenol‐1‐O‐[6‐O‐(3‐methoxy‐4‐hydroxybenzoyl)]‐β‐D‐glucopyranoside). The primary MS and cleavage patterns are illustrated in Figure 2I.
- Safflospermidine A/B (Compound 14): The UPLC retention time was 8.65 min. The compound exhibited a quasi‐molecular ion peak [M‐H]^−^ at *m/z* 582.2558, indicating a molecular weight of 583. A molecular formula of C_34_H_37_N_3_O_6_ was obtained after a comparative analysis with PubChem. Based on the secondary MS fragment ions (*m/z* 462.2020 [M‐C_8_H_8_O‐H]^−^, 342.1463 [M‐2C_8_H_8_O‐H]^−^, and 119.0495 [C_8_H_8_O‐H]^−^) and available literature [75, 76], the compound was identified as safflospermidine A/B. The secondary MS and cleavage patterns are illustrated in Figure 2J.
- Hemerocallal A (compound 28): The UPLC retention time was 19.95 min. The compound exhibited a quasi‐molecular ion peak [M‐H]^−^ at *m/z* 299.2014, indicating a molecular weight of 300. A molecular formula of C_20_H_28_O_2_ was obtained after a comparative analysis with PubChem. Based on the characteristic secondary MS fragment ion (*m/z* 149.0976) and available literature [77], the compound was identified as hemerocallal A. The secondary MS and cleavage pattern are illustrated in Figure 2K.

### 3.2 Blood and Tissue Distribution of the QSWZZP Components

**FIGURE 3:**
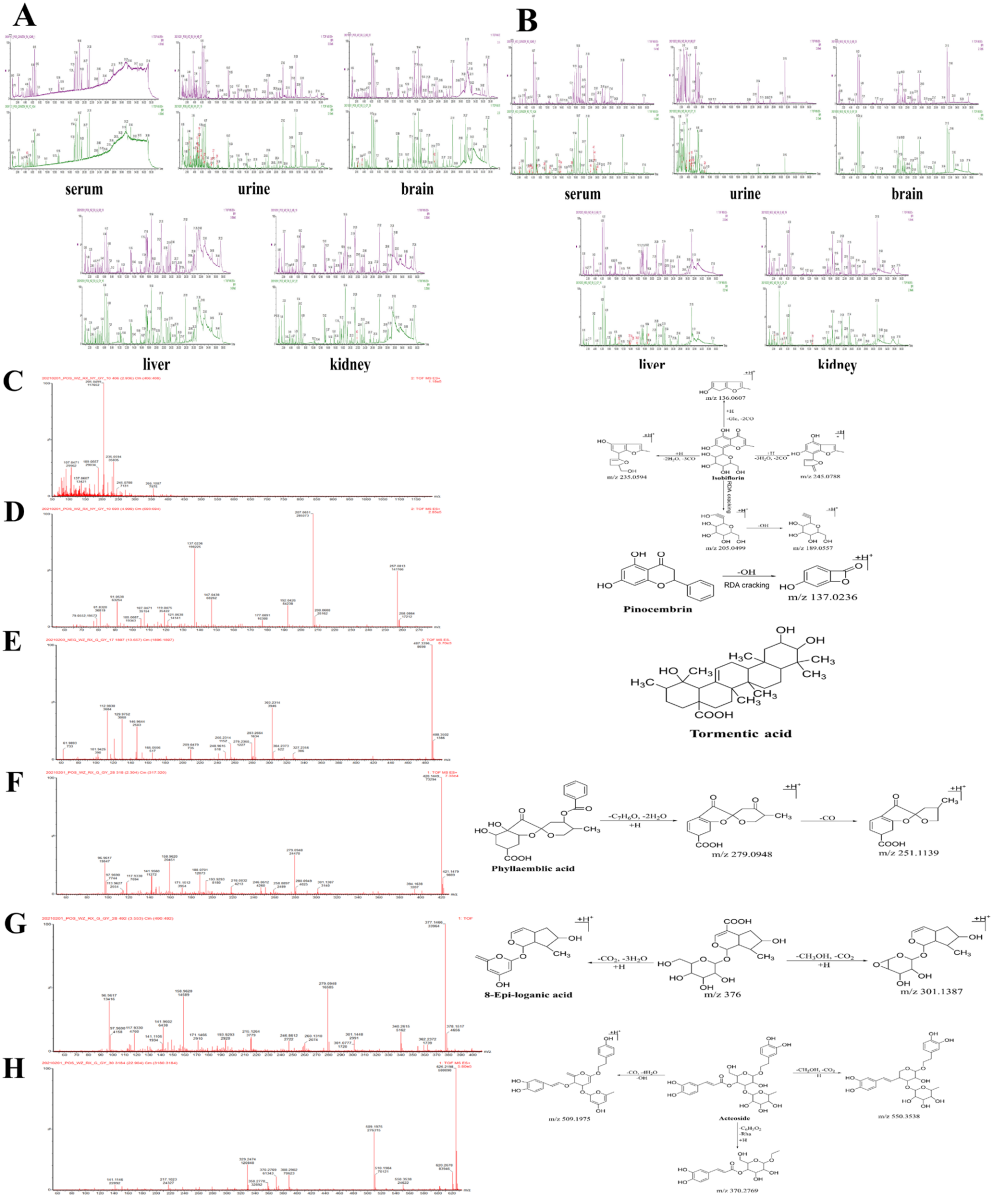
(A) Base‐peak ion flow (BPI) plots of the components distributed in the blood and tissues in the negative ion mode. (B) BPI plots of the components distributed in the blood and tissues in the positive ion mode non‐metabolite mass spectra and cleavage patterns/structural formulae. (C) Compound 6. (D) Compound 17. (E) Compound 33. (F) Compound 3. (G) Compound 38. (H) Compound 42.

Information on the identification of QSWZZP components and their blood and tissue distribution and the positive and negative BPI flow diagrams are presented in Table 2 and Figure 3A,B, respectively. By comparing the positive and negative BPI flow plots of the sham‐operated group with those of the model and QSWZZP groups, 45 components were identified. Fifteen were blood‐entry, 21 were urine‐entry, three were brain‐entry, seven were liver‐entry, and four were kidney‐entry components. Among these 45 components, 11 were described in previous experimental studies [18, 43], and were prototype products obtained in the present study as well. Among these, six components were distributed in the urine (quinic acid‐3‐caffeic acid ester (chlorogenic acid), syringin I, luteolin‐7‐O‐β‐D‐glucoside (luteoloside), agarotetrol, liquiritigenin, and 3′‐hydroxy‐8‐methoxyvestitol), three in the serum (glycyrrhizin, cholic acid, and arjunolic acid), two in the liver (cholic acid and arjunolic acid), and two in the kidneys (vanilloid and bear/goose/pig deoxycholic acid).

**TABLE 2:** Blood and tissue distribution of QSWZZP in rat model of cerebral ischemia: Component identification information.

Serial number	*t*_R_ (min)	Compounds	English name	Molecular formula	Ion mode	Fragmentation information (m/z)			Metabolic type	Distributions
						Parent ion		Daughter ion		
						Measured value	Theoretical value			
1	2.17	Chlorogenic acid^a^, ^b^	Chlorogenic acid	C_16_H_18_O_9_	[M‐H]^−^	353.0859	353.0873	275.0223,261.0444,233.0472,195.0667,150.0310	—	Urine
2	2.19	Reduction, methylation products of Syringaldehyde	Syringaldehyde	C_9_H_10_O_4_	[M‐H]^−^	181.0504	181.0501	—	Phase I and II metabolisms	Serum
3	2.30	Phyllaemblic acid	Phyllaemblic acid	C_21_H_24_O_9_	[M + H] ^+^	421.1479	421.1499	279.0948, 251.1139	—	Brain
4	2.35	Apocynin^a^	Apocynin	C_9_H_10_O_3_	[M‐H]^−^	165.0556	165.0552	187.0353, 119.0481	—	Kidney
5	2.62	BiflorinI^a^	BiflorinI	C_16_H_18_O_9_	[M + H] ^+^	355.1007	355.1029	259.0595, 245.0788, 235.0594, 205.0499, 178.0596, 147.0438	—	Urine
6	2.94	Isomers of Isobiflorin I	Isobiflorin	C_16_H_18_O_9_	[M + H] ^+^	355.1007	355.1029	245.0788, 235.0594, 205.0499, 189.0557, 137.0607	—	Urine
7	3.47	Reduction products of caffeic acid	3,4‐dihydroxyphenylpropanol	C_9_H_12_O_3_	[M‐H]^−^	167.0693	167.0708	373.1508, 139.0772	Phase I metabolism	Urine
8	3.55	8‐Epi‐loganic acid	8‐Epi‐loganic acid	C_16_H_24_O_10_	[M + H] ^+^	377.1466	377.1448	399.1250, 301.1387, 279.0948	—	Brain
9	4.28	Hydrolysis, hydroxylation, and methylation products of 2‐methoxy‐1,4‐diphenol‐1‐O‐[6‐O‐(3‐methoxy‐4‐hydroxybenzoyl)]‐β‐D‐glucopyranoside	3,5‐Dimethoxy‐4‐hydroxypheny methyl‐O‐β‐D‐glucopyranoside	C_15_H_22_O_9_	[M‐H]^−^	345.1158	345.1186	169.0862, 247.0658	Phase I and II metabolisms	Urine
10	4.59	Reduction, oxidation and methylation products of cholic acid	3‐Oxo tirucalla‐7,9(11),24‐trien‐21‐oic acid	C_30_H_44_O_3_	[M + H] ^+^	453.3448	453.3369	475.3288	Phase I and II metabolisms	Serum
11	4.70	Luteoloside^a^, ^b^	Luteolin‐7‐O‐β‐D‐glucoside (Cynaroside)	C_21_H_20_O_11_	[M‐H]^−^	447.0892	447.0927	429.2099, 331.1180, 175.0229	—	Urine
12	4.79	Agarotetrol^a^, ^b^	Agarotetrol	C_17_H_18_O_6_	[M + H] ^+^	319.1181	319.1182	341.0993, 301.1082, 283.0943, 255.0993, 227.1064, 164.0459, 136.0492, 105.0687, 91.0538	—	Urine
13	4.86	Deglycosylation products of kaempferol‐3‐O‐beta‐rutinoside	Kaempferol 3‐O‐rhamnoside (Afzelin)	C_21_H_20_O_10_	[M‐H]^−^	431.1013	431.0978	453.0846, 255.0666, 135.0072, 119.0495	Phase I metabolism	Serum, Urine, Kidney
14	4.86	Liquiritigenin^a^	Liquiritigenin	C15H_12_O_4_	[M + H] ^+^	257.0813	257.0814	147.0438, 137.0236	—	Urine
15	4.94	Deglycosylation and demethylation products of physcion‐8‐O‐β‐D‐gentiobioside	Emodin‐8‐O‐β‐D‐glucopy‐ ranoside (Anthraglycoside B)	C_21_H_20_O_10_	[M‐H]^−^	431.1013	431.0978	255.0627, 135.0066, 119.0495	Phase I metabolism	Serum, Urine
16	4.99	Deglycosylation and acetylation products of physcion‐8‐O‐β‐D‐gentiobioside	—	C_24_H_24_O_11_	[M + H] ^+^	489.1374	489.1397	455.0923, 257.0813, 207.0651, 147.0438, 137.0236	Phase I and II metabolisms	Urine
17	5.00	Isomers of liquiritigenin	Pinocembrin	C_15_H_12_O_4_	[M + H] ^+^	257.0813	257.0814	137.0236	—	Urine
18	5.28	3’‐Hydroxy‐8‐methoxyvestitol^a^	3’‐Hydroxy‐8‐methoxyvestitol	C_17_H_18_O_6_	[M + H] ^+^	319.1181	319.1182	341.0993, 301.1082, 255.0993, 227.1064, 136.0492	—	Urine
19	5.42	Oxidation products of liquiritigenin	Daidzein	C_15_H_10_O_4_	[M‐H]^−^	253.0487	253.0501	135.0066, 117.0329	Phase I metabolism	Urine
20	5.80	Oxidation, hydroxylation and methylation products of liquiritigenin	Calycosin	C_16_H_12_O_5_	[M + H] ^+^	285.0750	285.0763	270.0525, 213.0569, 197.0581, 137.0236	Phase I and II metabolisms	Urine
21	6.00	Liquiritin^a^, ^b^	Liquiritin	C_21_H_22_O_9_	[M‐H]^−^	417.1207	417.1186	439.0883, 135.0480, 119.0495	—	Serum
22	6.74	Methylation and glycosylation products of ellagic acid	—	C_23_H_22_O_13_	[M + H] ^+^	507.1116	507.1139	529.0941, 437.2123, 331.0822, 297.1649, 289.0093	Phase II metabolism	Urine
23	6.75	Hydroxylation and methylation products of luteolin	Tricin	C_17_H_14_O_7_	[M + H] ^+^	331.0822	331.0818	297.1649, 153.0907	Phase I and II metabolisms	Urine
24	7.19	Deglycosylation, hydroxylation and methylation products of physcion‐8‐O‐β‐D‐gentiobioside	Obtusin	C_18_H_16_O_7_	[M‐H]^−^	343.0844	343.0818	709.5117	Phase I and II metabolisms	Serum
25	8.59	Deglycosylation, hydroxylation, methylation and acetylation products of kaempferol‐3‐O‐β‐rutinoside	—	C_25_H_26_O_13_	[M + H] ^+^	535.1488	535.1452	557.1263, 475.2002, 359.1100, 125.0604	Phase I and II metabolisms	Urine
26	8.62	Hydrolysis, reduction, dehydroxylation, methylation and oxidation products of 2‐methoxy‐1,4‐diphenol‐1‐O‐ [6‐O‐ (3‐methoxy‐4‐hydroxybenzoyl)] ‐β‐D‐glucopyranoside	Picrocrocin	C_16_H_26_O_7_	[M‐H]^−^	329.1612	329.1600	312.1273, 269.1356	Phase I and II metabolisms	Urine
27	9.13	Demethylation and dehydroxylation products of 6‐methoxy‐2‐ (2‐ (3‐methoxyphenyl) ethyl) chromone	6‐Hydroxy‐2‐(2‐phenylethyl) chromone	C_17_H_14_O_3_	[M + H] ^+^	267.1003	267.1021	176.0465, 91.0538	Phase I metabolism	Urine
28	9.32	Reduction, oxidation and methylation products of cholic acid	(23R)‐3,7‐Dioxo‐tirucalla‐8,24‐dien‐21,23‐olide	C_30_H_42_O_4_	[M‐H]^−^	465.3018	465.3005	—	Phase I and II metabolisms	Serum
29	10.01	Deglycosylation and hydroxylation products of 1,7‐dimethoxy‐2,8‐dihydroxy‐3‐methylanthraquinone‐2‐O‐β‐D‐glucoside	Aurantioobtusin	C_17_H_14_O_7_	[M + H] ^+^	331.0822	331.0818	279.0948	Phase I metabolism	Urine
30	10.70	Cholic acid^a^	Hyocholic acid	C_24_H_40_O_5_	[M‐H]^−^	407.2818	407.2797	279.2376	—	Serum, Liver
31	13.03	Bear/goose/porcine deoxycholic acid^a^	Ursodeoxycholic acid/Chenodeoxycholic acid/Hyodeoxycholic acid	C_24_H_40_O_4_	[M‐H]^−^	391.2877	391.2848	437.2903	—	Kidney
32	13.37	Oxidation products of Hemerocallal A	(1R,4S,5R,6R,7R,13S,15E,17Z)‐4β‐Hydroxy‐15‐(3‐carboxyl‐2‐butenyl)‐aromadendr‐10(12),15‐dien	C_20_H_28_O_3_	[M‐H]^−^	315.1948	315.1960	—	Phase I metabolism	Serum
33	13.66	Isomers of Arjunic acid	Tormentic acid	C_30_H_48_O_5_	[M‐H]^−^	487.3396	487.3423	—	—	Liver
34	13.67	Arjunic acid^a^	Arjunic acid	C_30_H_48_O_5_	[M‐H]^−^	487.3448	487.3423	391.2907	—	Serum, Liver
35	13.69	Deglycosylation and acylation products of kaempferol‐3‐O‐β‐rutinoside	Kaempferol‐3‐O‐6″‐trans‐coumaroyl‐β‐D‐glucopyranoside	C_30_H_26_O_13_	[M‐H]^−^	593.1345	593.1295	283.2664	Phase I and II metabolisms	Liver
36	15.76	Hydroxylation and reduction products of luteolin	(−)‐Epigallocatechin	C_15_H_14_O_7_	[M‐H]^−^	305.0667	305.0661	255.2370	Phase I metabolism	Liver
37	16.05	Reduction and demethylation products of linoleic acid	Tetradecanoic acid	C_14_H_28_O_2_	[M‐H]^−^	227.2009	227.2011	—	Phase I metabolism	Liver
38	16.94	Reduction and demethylation products of linoleic acid	Palmitoleic acid	C_16_H_30_O_2_	[M‐H]^−^	253.2151	253.2168	—	Phase I metabolism	Serum
39	18.77	Reduction products of linoleic acid	Oleic acid	C_18_H_34_O_2_	[M‐H]^−^	281.2500	281.2480	255.2314	Phase I metabolism	Liver
40	22.03	Oxidation products of linoleic acid	α‐Linolenic acid	C_18_H_30_O_2_	[M + H] ^+^	279.2300	279.2324	261.2217	Phase I metabolism	Kidney
41	22.17	Reduction and methylation products of linoleic acid	Nonadecylic acid	C_19_H_38_O_2_	[M‐H]^−^	297.2808	297.2794	279.2317	Phase I and II metabolisms	Serum
42	22.90	Acteoside	Acteoside	C_29_H_36_O_15_	[M + H] ^+^	625.2198	625.2132	550.3538, 509.1975, 370.2769	—	Brain
43	23.18	Reduction and methylation products of vanilla acetophenone	Methyl thymyl ether	C_11_H_16_O	[M‐H]^−^	163.1114	163.1123	—	Phase I and II metabolisms	Serum
44	23.33	Reduction, oxidation and methylation products of linoleic acid	Arachidonic acid	C_20_H_32_O_2_	[M‐H]^−^	303.2327	303.2324	629.4584	Phase I and II metabolisms	Serum
45	23.94	Reduction and methylation products of linoleic acid	Arachidic acid	C_20_H_40_O_2_	[M‐H]^−^	311.2956	311.2950	283.2629	Phase I and II metabolisms	Serum

^a^ Prototype product.

^b^ Comparison with reference substance.

#### 3.2.1 Identification of Prototype Components and Non‐Metabolites Distributed in the Blood and Tissues

Excluding the eleven prototype components, six non‐metabolites distributed in the blood and tissues were identified, including three isomers of the prototype components (compounds 6, 17, and 33). Compound 6 (UPLC retention time of 2.94 min. positive ion mode) had a similar molecular formula, C_16_H_18_O_9_, as the prototype component butyroside I. Based on the similar fragmentation information of both ions [M‐2H_2_O‐3CO]^+^, the secondary MS fragment ions (*m/z* 245.0788 [M + H‐3H_2_O‐2CO]^+^ and 137.0607 [M + H‐Glc‐2CO]^+^), RDA cleavage fragment ions (*m/z* 205.0499 and 189.0557), and available literature [64], compound 6 was identified as an isomer of syringin I (syringin II). The secondary MS and cleavage patterns are illustrated in Figure 3C. Similarly, compound 17 (UPLC retention time of 5.00 min) had a similar molecular formula as liquiritigenin (C_15_H_12_O_4_). Based on the characteristic RDA fragment ions (*m/z* 137.0236 and 135.0112) and available literature [78, 79], compound 17 was identified as pinocembrin. The secondary MS and cleavage patterns are illustrated in Figure 3D. Compound 33 was identified as an isomer of arjunolic acid based on the available literature [80, 81], and its secondary MS and structural formula are illustrated in Figure 3E.

In addition, three non‐metabolites distributed in the brain tissue, such as amlaic acid, 8‐epi‐loganic acid, and acteoside (compounds 3, 8 and 42, respectively), were identified based on available literature [82, 83]. Their primary MS and cleavage patterns are illustrated in Figure 3F–H.

#### 3.2.2 Identification of Metabolites Distributed in the Blood and Tissues

Twenty‐eight metabolites distributed in the blood and tissues were identified: twelve phase Ι metabolites, one phase II metabolite, and fifteen phase Ι and II co‐metabolites. Phase Ι metabolism mainly comprises oxidation, reduction, hydrolysis, hydroxylation, deglycosylation, and demethylation reactions, whereas phase II metabolism is mainly dominated by binding reactions, such as glucuronide, amino acid, glutathione, sulfation, acetylation, and methylation binding [84, 85].

Among the prototype compounds of the metabolites obtained, organic acids were the majority, followed by flavonoids, steroids, anthraquinones, phenols, chromones, triterpenes, and other compounds. The quasi‐molecular ion peaks [M + H]^+^, [M + Na]^+^ and [M‐H]^−^, [M + Na‐2H]^−^, [2M + Na‐2H]^−^, and [M + HCOOH‐H]^−^ of most metabolites were observed. Simultaneously, the loss of secondary MS ions ^−^CH_3_ (15 Da) and ^−^OH (17 Da) indicated a possible methylation and hydroxylation product of a prototype compound. In addition, the prototype component was deglycosylated or glycosylated. If the glucosyl group (‐C_6_H_10_O_5_, 162 Da) or rhamnosyl group (‐C_6_H_10_O_4_, 146 Da) was lost or obtained, the corresponding metabolites could be formed as well.

##### 3.2.2.1 Organic Acid Metabolites

Organic acid metabolites are important components distributed in the blood and tissues. Their main MS features are characteristic fragment ions such as [M‐H‐CO]^−^, [M‐H‐CO_2_]^−^, [M‐H‐H_2_O]^−^, [M‐CH_3_]^−^, and [M + H‐H_2_O]^+^.

Based on the aforementioned MS characteristics, information on elemental composition, retention time, and secondary MS fragmentation, 10 organic acid metabolites were identified in the biological samples after modeling and drug administration: five were distributed in the serum, two in the urine, two in the liver, and one in the kidneys. The metabolism mainly comprised reduction, oxidation, hydroxylation, and demethylation in phase Ι combined with glycosylation and methylation in phase II. The prototypical organic acid metabolite components identified were mainly linoleic acid, followed by gallic acid, caffeic acid, and ellagic acid.

**FIGURE 4:**
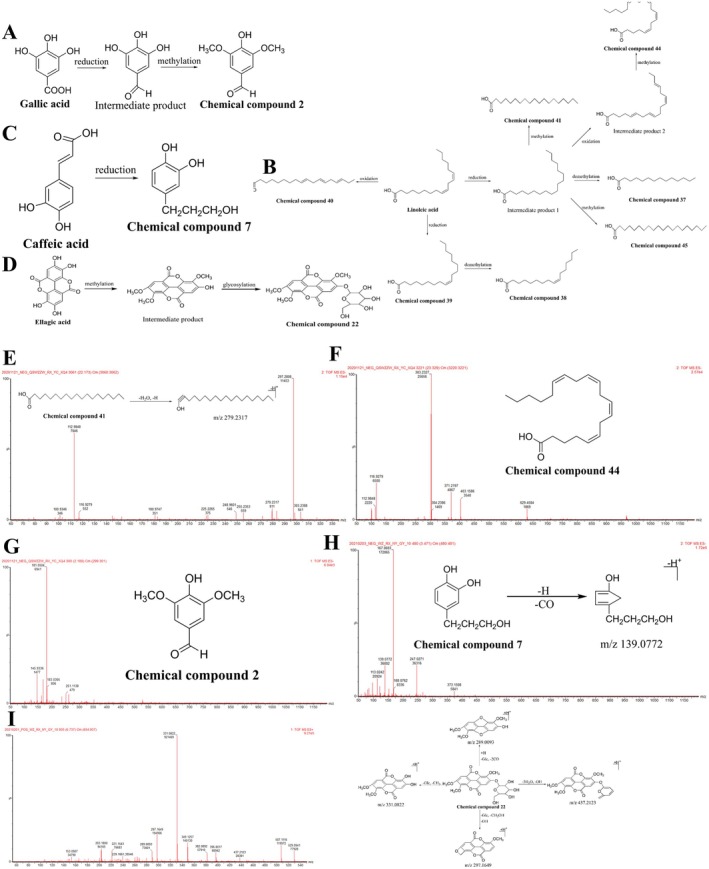
Metabolic pathways of organic acids and their metabolites with mass spectra and cleavage patterns/structural formulae. (A) Gallic acid. (B) Linoleic acid. (C) Caffeic acid. (D) Ellagic acid. (E) Linoleic acid metabolite: Compound 41. (F) Linoleic acid metabolite: Compound 44. (G) Gallic acid metabolite: Compound 2. (H) Caffeic acid metabolite: Compound 7. (I) Ellagic acid metabolite: Compound 22.

Seven metabolites were identified with linoleic acid as the prototype, and they were inferred to undergo oxidation, reduction, and methylation in vivo. Compounds 41 and 44 were considered as examples, based on the molecular formulae (C_19_H_38_O_2_ and C_20_H_32_O_2_) calculated using the MS analysis software, the quasi‐molecular ion peaks (*m/z* 297.2808 [M‐H]^−^, 303.2327 [M‐H]^−^, and 629.4584 [2 M + Na‐2H]^−^), the MS fragment ions (*m/z* 279.2317 [M‐H_2_O‐H]^−^), and available literature [86, 87]; the compounds were identified as 19‐alkanoic acid and arachidonic acid. The secondary MS and cleavage patterns are illustrated in Figure 4E,F. While 19‐alkanoic acid is a reduction and methylation product of linoleic acid, arachidonic acid is a reduction, oxidation, and methylation product of linoleic acid. The remaining five linoleic acid metabolites (compounds 37–40 and 45) were similarly formed. The linoleic acid metabolic pathway is illustrated in Figure 4B.

The gallic acid metabolite, with a UPLC retention time of 2.19 min, exhibited a quasi‐molecular ion peak [M‐H]^−^ at *m/z* 181.0504, and a molecular formula of C_9_H_10_O_4_ was identified using MS analysis software and available literature [88]. Compound 2 was identified as syringaldehyde, and the primary MS pattern and structural formula are illustrated in Figure 4G. The metabolic pathway of gallic acid in vivo is depicted in Figure 4A. Similarly, compound 7 was identified as the reduction product of caffeic acid [84]. The secondary MS and cleavage patterns are illustrated in Figure 4H. The metabolic pathway of caffeic acid in vivo is depicted in Figure 4C.

One ellagic acid metabolite was identified. Compound 22 (UPLC retention time of 6.74 min) exhibited quasi‐molecular ion peaks at *m/z* 507.1116 [M + H]^+^ and 529.0941 [M + Na]^+^, and a molecular formula of C_23_H_22_O_13_ was identified. Moreover, based on the primary MS fragment ion (*m/z* 331.0822 [M + H‐Glc‐CH_2_]^+^) with a molecular formula of C_16_H_10_O_8_ and available literature, the compound was identified as 3,3′,4‐trimethyl ellagic acid‐4′‐O‐β‐D‐glucoside [64]. The primary MS and cleavage patterns are illustrated in Figure 4I. The metabolic pathway of ellagic acid in vivo is depicted in Figure 4D.

##### 3.2.2.2 Flavonoid Metabolites

**FIGURE 5:**
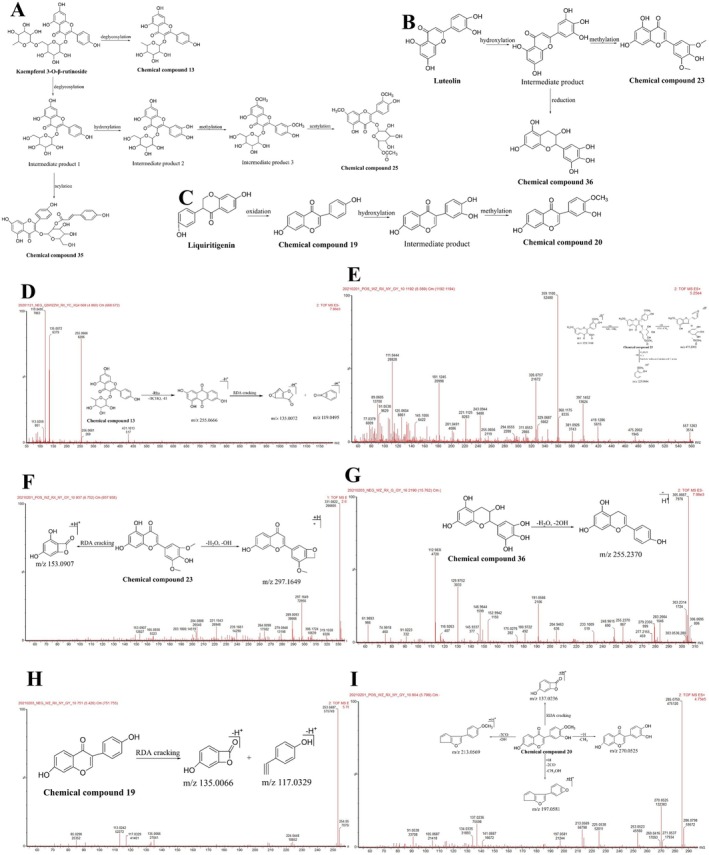
Metabolic pathways of flavonoids and the mass spectra and cleavage patterns of their metabolites. (A) Kaempferol‐3‐O‐β‐rutinoside. (B) Luteolin. (C) Liquiritigenin. (D) Kaempferol‐3‐O‐β‐rutinoside metabolite: Compound 13. (E) Kaempferol‐3‐O‐β‐rutinoside metabolite: Compound 25. (F) Luteolin metabolite: compound 23 (G) Luteolin metabolite: compound 36. (H) Liquiritigenin metabolite: compound 19. (I) Liquiritigenin metabolite: compound 20.

Flavonoid metabolites are an important class of metabolites found distributed in the blood and tissues. Flavonoids are often linked to various sugar groups to form flavonoid glycosides. The primary/secondary MS of these metabolites often contains fragments of the glucosyl group (‐Glc, 162 Da) and rhamnosyl group (‐Rha, 146 Da), and the characteristic MS fragment ions are *m/z* 285.0426 [M‐Rha‐Glc‐H]^−^ and 255.0666 [M‐Rha‐HCHO‐H]^−^. Flavonoids are prone to RDA cleavage to form characteristic fragment ions of different sizes (e.g., *m/z* 135.0072, 119.0495, etc.). Based on the aforementioned secondary MS characteristics and the available information in the literature on the elemental composition, retention time, and secondary MS fragment ions, seven flavonoid metabolites were identified: one in the serum, four in the urine, two in the liver, and one in the kidneys. The flavonoid metabolites underwent phase Ι and phase II metabolism via oxidation, reduction, hydroxylation, deglycosylation, methylation, and acetylation [84, 85] (Table 2). Among the flavonoid metabolites, three were kaempferol‐3‐O‐β‐rutinoside, two were luteolin, and two were liquiritigenin metabolites. The specific process is described herewith:

Three kaempferol‐3‐O‐β‐rutinoside metabolites were identified (compound 13). Compound 13 (UPLC retention time of 4.86 min) exhibited a quasi‐molecular ion peak at *m/z* 431.1013 [M‐H]^−^ and 453.0846 [M + Na‐2H]^−^, and its molecular formula was C_21_H_20_O_10_. Based on the fragment ions (*m/z* 255.0666 [M‐Rha‐HCHO‐H]^−^, 135.0072, and 119.0495) and the characteristic products of RDA cleavage, compound 13 was deduced to be a flavonoid metabolite. Based on analyses and available literature [89], compound 13 was identified as kaempferol‐3‐O‐rhamnoside. The secondary MS and cleavage patterns are illustrated in Figure 5D. Based on analyses and available literature [64] as well, compound 25 was identified as rhamnetin‐3‐O‐β‐D‐(6″‐acetyl)glucoside. The secondary MS and cleavage patterns are illustrated in Figure 5E. Compound 35 was similarly identified as the deglycosylation and acylation product of kaempferol‐3‐O‐β‐rutinoside. The metabolic pathway of kaempferol‐3‐O‐β‐rutinoside is illustrated in Figure 5A.

Two luteolin metabolites were identified (compounds 23 and 36). Based on the characteristic primary MS ion (*m/z* 153.0907) of RDA cleavage, the molecular formula (C_17_H_14_O_7_) of compound 23 was obtained, and it was identified as the hydroxylation and methylation product of luteolin. Based on the primary MS fragment ion (*m/z* 297.1649) and available literature [83], the compound was identified as wheat flavin, and its primary MS and cleavage patterns are illustrated in Figure 5F. Similarly, compound 36 was deduced as the reduction and hydroxylation product of luteolin and was identified as (−)‐epigallocatechin based on available literature [89]. The secondary MS and cleavage patterns are illustrated in Figure 5G. The metabolic pathway of luteolin in vivo is depicted in Figure 5B.

Two liquiritigenin metabolites were identified (compounds 19 and 20). Based on the aforementioned analysis method and available literature [9, 30, 90, 91], compound 19 (C_15_H_10_O_4_) was identified as daidzein, and its secondary MS and cleavage patterns are illustrated in Figure 5H. Compound 20 (C_16_H_12_O_5_) was similarly identified as calycosin, and its secondary MS and cleavage patterns are illustrated in Figure 5I. The metabolic pathway of liquiritigenin is depicted in Figure 5C.

##### 3.2.2.3 Anthraquinone Metabolites

The formation mechanism of anthraquinone metabolites is similar to that of flavonoid metabolites. The anthraquinone parent nucleus is often linked to a sugar group to form anthraquinone derivatives, which easily remove the sugar group in vivo. The MS of such metabolites may contain fragments of the glucosyl (‐Glc, 162 Da) and rhamnosyl (Rha, 146 Da) groups, such as *m/z* 255.0627 [M‐Glc‐CH_3_]^−^. The anthraquinone parent core is readily co‐cleaved with the hydroxyl group attached to it to remove CO and CO_2_, thereby forming the characteristic [M‐CO] and [M‐CO2] fragments [92, 93]. In addition, anthraquinone metabolites undergo RDA cleavage to form different fragment ions. Accordingly, in this study, four anthraquinone metabolites were identified as the prototypical components, two in serum and three in urine, which mainly underwent phase‐Ι and II metabolism. While three metabolites were metabolized by physcion diglucoside, physcion 8‐gentiobioside, and physcion‐8‐O‐β‐gentiobioside, one metabolite was metabolized by 1,7‐dimethoxy‐2,8‐dihydroxy‐3‐methyl‐anthraquinone‐2‐O‐β‐D‐1, a glucoside metabolite.

**FIGURE 6:**
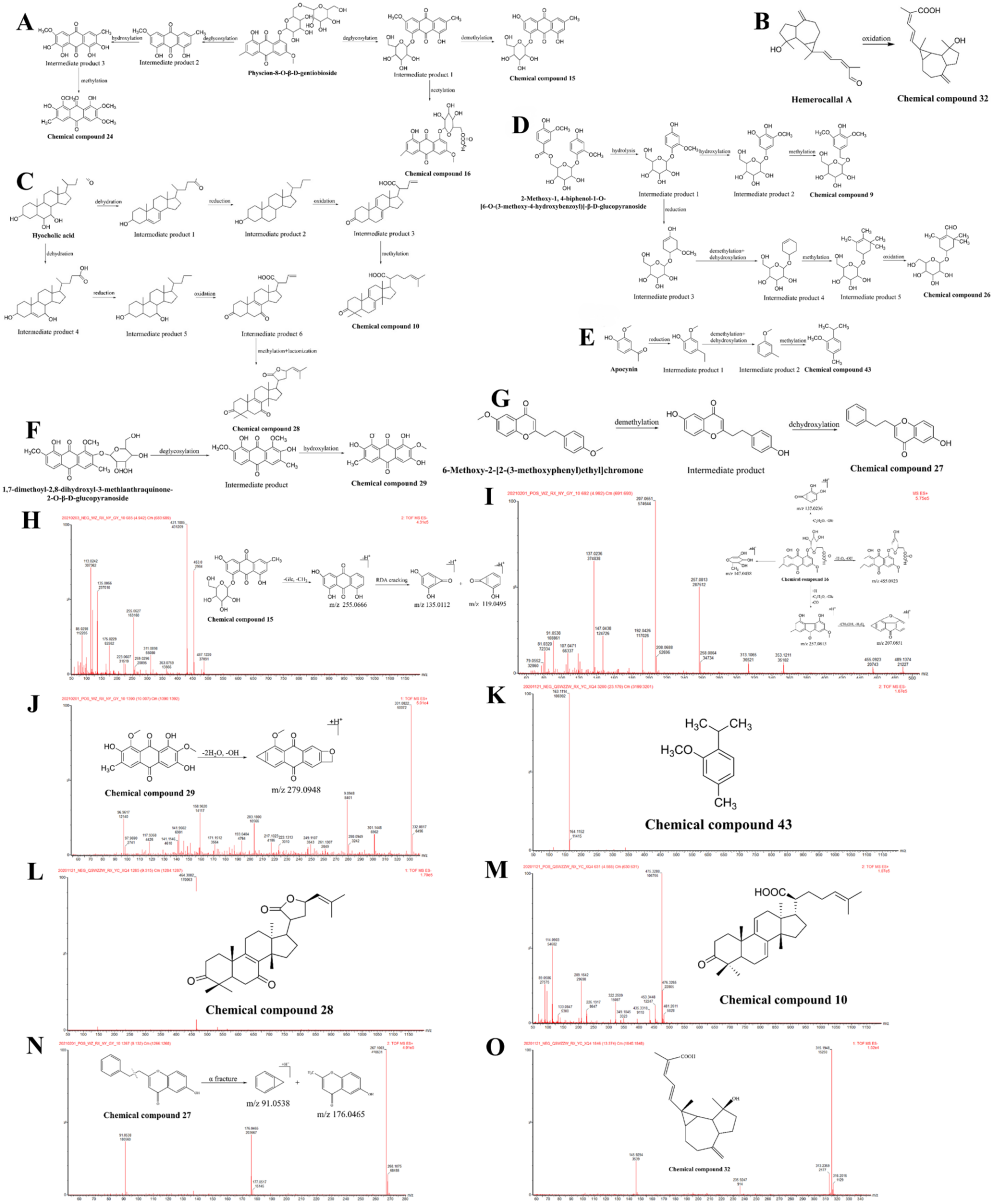
Metabolic pathways of anthraquinones, phenols, steroids, chromones, and other compounds and their metabolites with mass spectra and cleavage patterns (A) Physcion‐8‐O‐β‐D‐gentiobioside metabolic pathway. (B) Hemerocallal A metabolic pathway. (C) Cholic acid metabolic pathway. (D) Phenolic components metabolic pathway. (E) Apocynin metabolic pathway, showing key intermediates. (F) 1,7‐dimethoxy‐2,8‐dihydroxy‐3‐methylanthraquinone‐2‐O‐β‐D‐glucoside metabolic pathway. (G) 6‐methoxy‐2‐ (2‐(3‐methoxyphenyl)ethyl) chromone metabolic pathway. (H) Physcion‐8‐O‐β‐D‐gentiobioside metabolite: compound 15. (I) Physcion‐8‐O‐β‐D‐gentiobioside metabolite: compound 16. (J) 1,7‐dimethoxy‐2,8‐dihydroxy‐3‐methylanthraquinone‐2‐O‐β‐D‐glucoside metabolite: compound 29. (K) Phenolic metabolite: compound 43. (L) Steroid metabolite: compound 28. (M) Steroid metabolite: compound 10. (N) Chromone metabolite: compound 27. (O) Other metabolite: compound 32.

Three physcion‐8‐O‐β‐D‐gentiobioside metabolites were identified (compounds 15, 16, and 24). Compound 15 (UPLC retention time of 4.99 min) exhibited a quasi‐molecular ion peak at *m/z* 431.1013 [M‐H]^−^. It was deduced as the deglycosylation and demethylation product of physcion‐8‐O‐β‐D‐gentiobioside. Based on the secondary MS, the fragment ion peaks (*m/z* 399.0564 [M‐CH_3_‐H_2_O]^−^ and 319.0932 [M‐CH_2_OH‐3H_2_O‐CO]^−^), fragment ion *m/z* 255.0666 [M‐Glc‐CH_3_]^−^, fragment peaks (*m/z* 135.0112 and *m/z* 119.0495) and available literature [94], the compound was identified as emodin‐8‐O‐β‐D‐glucoside after a comparative analysis of the structure and molecular formula of the prototype component. The secondary MS and cleavage patterns are illustrated in Figure 6H. Compound 16 (UPLC retention time of 4.99 min) exhibited an excimer ion peak at *m/z* 489.1374 [M + H]^+^ and a fragment ion, *m/z* 257.0813 [M + H‐C_2_H_2_O‐Glc‐CO]^+^. Accordingly, it was identified as the deglycosylation and acetylation product of physcion‐8‐O‐β‐D‐gentiobioside after a comparative analysis. The secondary MS and cleavage patterns are illustrated in Figure 6I. Compound 24 was identified as the deglycosylation, hydroxylation, and methylation product of physcion‐8‐O‐β‐D‐gentiobioside. The metabolic pathway of physcion‐8‐O‐β‐D‐gentiobioside is depicted in Figure 6A. Compound 29 (UPLC retention time of 10.01 min) exhibited a quasi‐molecular ion peak at *m/z* 331.0822 [M + H]^+^, and its molecular formula was identified as C_17_H_14_O_7_. The prototype component was hypothesized to have first removed one glucosyl (‐C_6_H_10_O_5_) group and subsequently hydroxylated to form compound 29. Based on available literature [95, 96], the compound was identified as aurantio obtusin (a 1,7‐dimethoxy‐2,8‐dihydroxy‐3‐methylanthraquinone‐2‐O‐β‐D‐glucoside metabolite). The primary MS and cleavage pattern are illustrated in Figure 6J. The metabolic pathway of 1,7‐dimethoxy‐2,8‐dihydroxy‐3‐methylanthraquinone‐2‐O‐β‐D‐glucoside is depicted in Figure 6F.

##### 3.2.2.4 Phenolic Metabolites

Phenolic metabolites are more common following QSWZZP administration. Based on the analysis of the elemental composition of the compounds, secondary MS fragmentation information, and available literature, three phenolic metabolites were identified: one in serum and two in urine. As presented in Table 2, the phenolic metabolites were formed in vivo via phase I and II metabolism, involving reduction, hydroxylation, and methylation. The prototypical components were 2‐methoxy‐1, 4‐diphenol‐1‐O‐[6‐O‐(3‐methoxy‐4‐hydroxybenzoyl)]‐β‐D‐glucopyranoside and vanillylacetophenone. For example, compound 43 (UPLC retention time of 23.18 min) exhibited a quasi‐molecular ion peak [M‐H]^−^ at *m/z* 163.1114, and a molecular formula of C_11_H_16_O was identified. Since this was found to be near‐similar to that of vanillylacetophenone (C_9_H_10_O_3_), it was deduced as a reduction and methylation product of vanillylacetophenone based on an analysis and available literature [97]. Compound 43 was identified as thymol methyl ether, and its secondary MS and structural formula are illustrated in Figure 6K. Compounds 9 and 26 were homologous. The metabolic pathways of the phenolic components are depicted in Figure 6E.

##### 3.2.2.5 Steroid Metabolites

The prototype components of steroid metabolites are mainly bile acids, which are present in the MS as the quasi‐molecular ion peak [M‐H]^−^ at *m/z* 407.2818. They may undergo decarboxylation and dehydration to form the characteristic ion [M‐HCOOH‐H_2_O‐H]^−^ at *m/z* 343.2669. Accordingly, two bile acids were identified from the biological samples after QSWZZP administration, both of which were found in serum and mainly underwent phase‐Ι and II metabolism. The reduction, oxidation, and methylation pathways of bile acids produced compounds 10 and 28, with the related parent ions *m/z* 453.3448 [M + H]^+^, 475.3288 [M + Na]^+^, and 465.3018 [M‐H]^−^, respectively. Their MS and structural formulae are illustrated in detail in Figure 6L,M. The metabolic pathways of bile acids are depicted in Figure 6C.

##### 3.2.2.6 Chromone Metabolites

One chromone metabolite was identified (compound 27) in the urine, which mainly underwent phase I metabolism via demethylation. The prototype component was 6‐methoxy‐2‐(2‐(3‐methoxyphenyl) ethyl) chromone. Compound 27 (UPLC retention time of 9.13 min) exhibited a quasi‐molecular ion peak [M + H]^+^ at *m/z* 267.1003 and was identified to have a molecular formula of C_17_H_14_O. Based on the consonance between the two ion peaks (*m/z* 176.0465 and 177.0562) and available literature [73], compound 27 was identified as 6‐methoxy‐2‐(2‐phenylethyl). The secondary MS and cleavage patterns are illustrated in Figure 6N. The metabolic pathway of 6‐methoxy‐2‐(2‐(3‐methoxyphenyl) ethyl) chromone is depicted in Figure 6G.

##### 3.2.2.7 Other Metabolites

One metabolite of another compound class was identified (compound 32) in serum and mainly undergoing phase I metabolism. The prototype component was hemerocallal A, with a molecular formula of C_20_H_28_O_3_. Compound 32 (UPLC retention time of 13.37 min) exhibited a quasi‐molecular ion peak [M‐H]^−^ at *m/z* 315.1948. Based on a comparison with the molecular formula of the prototype component and available literature [98], compound 32 was identified as (1R,4S,5R,6R,7R. 13S,15E,17Z)‐4β‐hydroxy‐15‐(3‐carboxy‐2‐butenyl)‐vanillane‐10(12),15‐diene. The primary MS and structural formulae are illustrated in Figure 6O. The metabolic pathway of hemerocallal A is depicted in Figure 6B.

### 3.3 QSWZZP Differential Metabolites and Their Metabolic Pathways in Cerebral Ischemia Treatment

**FIGURE 7:**
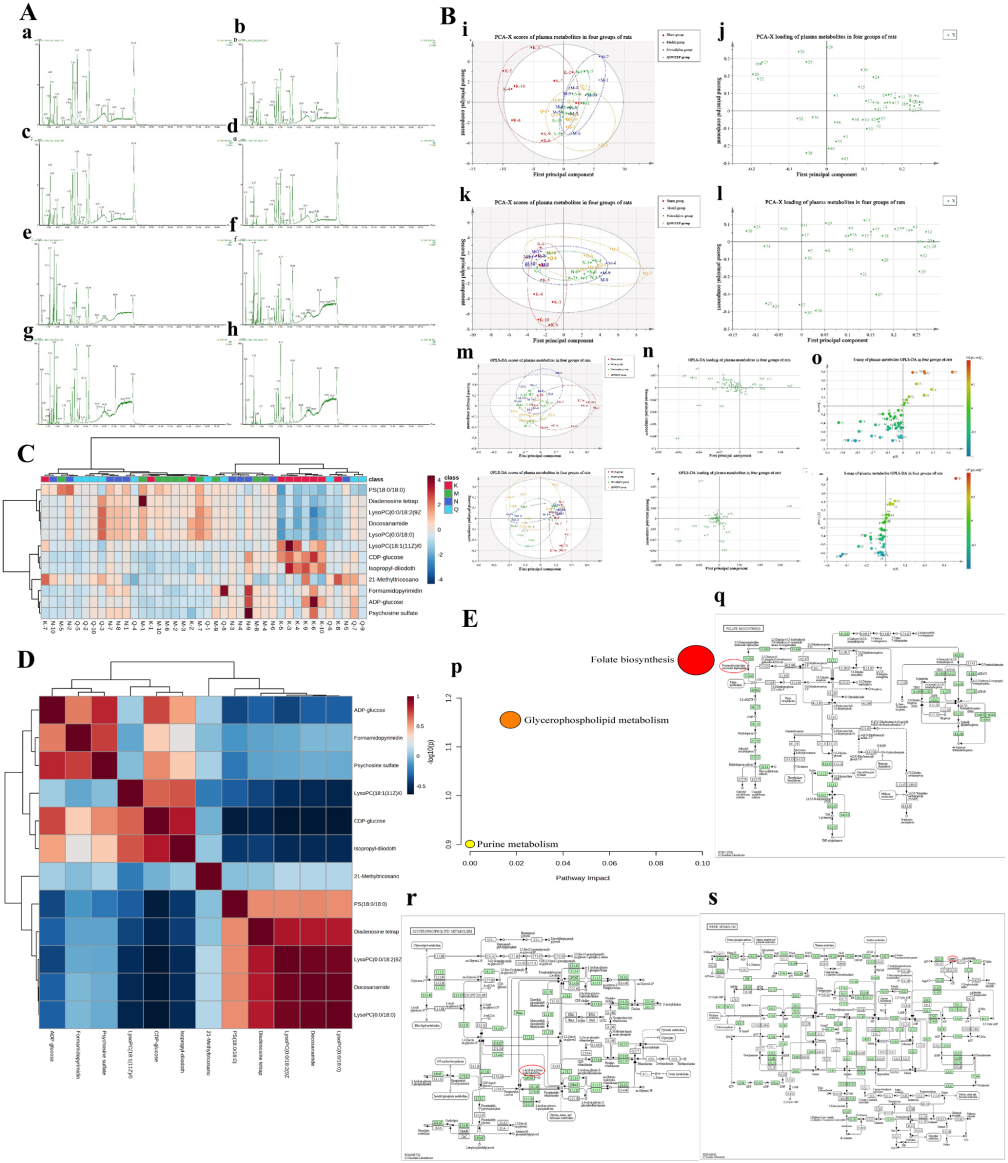
Differential metabolites and the metabolic pathway map of QSWZZP in cerebral ischemia treatment (A) UPLC‐Q‐TOF‐MS base‐peak ion current diagram of plasma metabolites in each rat group in the positive and negative ion modes (positive ion mode: (a) sham‐operated group, (b) model control group, (c). nimodipine control group, (d) QSWZZP group. negative ion mode: (e) sham‐operated group, (f) model control group, (g) nimodipine control group, (h) QSWZZP group). (B) PCA score plot and loading plot of plasma metabolites in four groups of rats in the positive and negative ion modes (positive ion mode: (i) score plot, (j) load plot. negative ion mode: (k) score plot, (l) load plot). (C) PCA score plot and load plot of plasma metabolites in four rat groups in the positive and negative ion modes (Positive and negative ion mode: (m) score plot, (n) load plot, (o) S‐plot). (D) Differential metabolite heatmap (s) content heatmap‐the horizontal axis indicates only the individual rat, where K refers to the sham‐operated group, M refers to the model control group, N refers to the nimodipine control group, and Q refers to the QSWZZP group. (t) Correlation heatmap. (E) Metabolic pathway diagrams ((p) bubble diagram of three metabolic pathways. (q) Folate biosynthesis pathway. (r) Glycerophospholipid metabolism pathway. (s) Purine metabolism pathway).

All plasma samples were analyzed to obtain representative positive and negative BPI flow chromatograms for each group (Figure 7A). The non‐volatile metabolic components in plasma after modeling and drug administration were identified and analyzed via the HMDB library search, and the relative percentage of each chromatographic peak alongside the relevant MS was analyzed and confirmed using the peak‐area normalization method.

#### 3.3.1 Principal Component Analysis (PCA)

In the positive ion mode, the plasma metabolites of the sham‐operated group and model control groups were significantly separated, indicating some between‐group differences in the metabolite components (Figure 7B). The plasma metabolites of the QSWZZP and nimodipine control and model control groups were not significantly separated. The PCA scoring plot model parameter was R2X = 0.441. As depicted in the loading plot (Figure 7B), the contribution by No. 27 (lysophosphatidylcholine [PC](18:1(11Z)/0:0)) was largest, followed by No. 19 (dCTP) and No. 20 (lysoPC(20:5(5Z,8Z,11Z,14Z,17Z)/0:0)), and the smallest was by No. 44 (PC(16:0/18:2(9Z,12Z))).

In the negative ion mode, the plasma metabolites of the sham‐operated and model control groups were clearly separated (Figure 7B). The plasma metabolites of the QSWZZP and model control groups were fairly separated, albeit not significantly, suggesting t some between‐group differences in the metabolic components. The plasma metabolites of the nimodipine control and model control groups were not clearly separated. The PCA scoring plot model parameter was R2X = 0.411. As depicted in the loading diagram (Figure 7B), the contribution of No. 29 (psychosine sulfate) was the largest, followed by No. 32 (lysoPC (22:5(4Z,7Z,10Z,13Z,16Z)/0:0)) and No. 23 (GDP‐4‐dehydro‐6‐L‐deoxygalactose), and the smallest was by No. 44 (hyodeoxycholic acid).

#### 3.3.2 Orthogonal Partial Least Squares Discriminant Analysis (OPLS‐DA)

In the positive ion mode, the plasma metabolites of the nimodipine control and model control groups were not significantly separated, whereas the plasma metabolites of the QSWZZP and sham‐operated and model control groups were significantly separated (Figure 7C), indicating significant between‐group differences in the metabolic components. The plasma metabolites of the nimodipine control and QSWZZP groups were similar and were distinct from those of the sham‐operated group. The OPLS‐DA model parameters were R2X = 0.384, R2Y = 0.296, and Q2 = 0.153. The loading diagram (Figure 7C) revealed that highly correlated metabolites were clustered together, such as No. 6 (docosanamide) and No. 29 (lysoPC(0:0/18:0)), whereas the uncorrelated metabolites were clustered on both ends of the lines passing through the origin, such as No. 23 (lysoPC(0:0/18:2(9Z,12Z))) and No. 27 (lysoPC(18:1(11Z)/0:0)). The S‐plot (Figure 7C) revealed that No. 27 (lysoPC(18:1(11Z)/0:0)) and No. 29 (lysoPC(0:0/18:0)), which had the largest difference and variable importance in the projection (VIP) values, may be potential biomarkers or differential metabolites.

In the negative ion mode, the plasma metabolites of the sham‐operated, nimodipine control, QSWZZP, and model control groups were fairly separated (Figure 7C), indicating some between‐group differences in the metabolite components. The plasma metabolites of the nimodipine control and QSWZZP groups were similar and were distinct from those of the sham‐operated group. The OPLS‐DA model parameters were R2X = 0.719, R2Y = 0.314, and Q2 = 0.138. The loading diagram (Figure 7C) revealed that uncorrelated metabolites, such as No. 29 (psychosine sulfate) and No. 37 (5‐thia‐1‐azabicyclo[4.2.0]oct‐2‐ene‐2‐carboxylic acid, 3‐[[(aminocarbonyl)oxy]methyl]‐7‐[[(2Z)‐2‐furanyl(methoxyimino)acetyl]amino]‐8‐oxo‐, (6R,7R)), were clustered at the ends of the line passing through the origin. The S‐plot (Figure 7C) revealed that No. 32 (lysoPC(22:5(4Z,7Z,10Z,13Z,16Z)/0:0)) and No. 38 (3alpha,7alpha‐dihydroxycoprostanic), which had the largest values of difference and VIP, may be potential biomarkers or differential metabolites.

#### 3.3.3 Differential Metabolites

OPLS‐DA was used to analyze the plasma metabolites in each experimental group. When the VIP was > 1, the metabolite was confirmed as a potential biomarker. The normality test revealed that the data conformed to the normal distribution (*p* > 0.05). Hence, the potential biomarkers were analyzed using the one‐way ANOVA. When *p* < 0.05, the metabolite was identified as a differential metabolite.

In the positive ion mode, 10 potential biomarkers and seven differential metabolites were identified from the 44 plasma metabolites identified. The differential metabolites were docosanamide, PS(18:0/18:0), lysoPC(0:0/18:2(9Z,12Z)), lysoPC(18:1(11Z)/0:0), lysoPC(0:0/18:0), 21‐methyltricosanoylcarnitine, and diadenosine tetraphosphate(AppppA). Two of these (lysoPC(18:1(11Z)) and diadenosine tetraphosph) had a Kyoto Encyclopaedia of Genes and Genomes (KEGG) annotation numbering (Table 3). The model control group had significantly higher docosanamide, PS (18:0/18:0), lysoPC (0:0/18:2(9Z,12Z)), and lysoPC (0:0/18:0) (all *p* < 0.01) and diadenosine tetraphosphate (*p* < 0.05) levels and significantly lower lysoPC (18:1(11Z)/0:0) levels (*p* < 0.05) than the sham‐operated group. 21‐methyltricosanoylcarnitine levels were significantly higher in the nimodipine control group than in the model control group (*p* < 0.05). PS (18:0/18:0) levels in the QSWZZP group were significantly decreased (*p* < 0.01), similar to that in the sham‐operated group. The levels of other metabolites, including lysoPC (18:1(11Z)/0:0), were significantly decreased (*p* < 0.05).

**TABLE 3:** Differential metabolite‐related information in the positive/negative ion mode (*n* = 10, $\bar{x}\pm s$).

Ion mode	Serial number	*VIP* value	Compounds	HMDB number	KEGG number	Molecular formula	Sham operation group	Model group	Nimodipine control group	Qishiwei Zhenzhu pills group
[M + H] ^+^	6	1.6975	Docosanamide	HMDB0000583	—	C_22_H_45_NO	0.0396 ± 0.0105**	0.0513 ± 0.0061	0.0512 ± 0.0055	0.0514 ± 0.0081
	7	1.8539	PS (18:0/18:0)	HMDB0012378	—	C_42_H_82_NO_10_P	0.0453 ± 0.0132**	0.0667 ± 0.0100	0.0631 ± 0.0119	0.0505 ± 0.0081**
	23	1.9259	LysoPC (0:0/18:2(9Z,12Z))	HMDB0061700	—	C_26_H_50_NO_7_P	0.0513 ± 0.0125**	0.0681 ± 0.0078	0.0673 ± 0.0072	0.0675 ± 0.01058
	27	1.6054	LysoPC (18:1(11Z)/0:0)^#^	HMDB0010385	C04230	C_26_H_52_NO_7_P	0.02453 ± 0.0150*	0.0114 ± 0.0019	0.0117 ± 0.0032	0.0086 ± 0.0027*
	29	1.6613	LysoPC (0:0/18:0)	HMDB0011128	—	C_26_H_54_NO_7_P	0.0370 ± 0.0088**	0.0493 ± 0.0057	0.0487 ± 0.0052	0.0489 ± 0.0077
	35	1.3334	21‐Methyltricosanoylcarnitine	HMDB0240986	—	C_31_H_61_NO4	0.0783 ± 0.0459	0.0605 ± 0.0146	0.0805 ± 0.0213*	0.0787 ± 0.0263
	39	1.1650	Diadenosine tetraphosphate^#^	HMDB0001211	C01260	C_20_H_28_N_10_O_19_P_4_	0.0168 ± 0.0039*	0.0239 ± 0.0069	0.0220 ± 0.0024	0.0219 ± 0.0033
[M‐H]^−^	26	1.3650	CDP‐glucose^#^	HMDB0003369	C00501	C_15_H_25_N_3_O_16_P_2_	0.0191 ± 0.0086**	0.0103 ± 0.0028	0.0131 ± 0.0043	0.0103 ± 0.0029
	27	1.3352	ADP‐glucose^#^	HMDB0006557	C00498	C_16_H_25_N_5_O_15_P_2_	0.0299 ± 0.0178	0.0216 ± 0.0050	0.0295 ± 0.0101*	0.0241 ± 0.0064
	28	1.3379	Formamidopyrimidine nucleoside triphosphate^#^	HMDB0006822	C05922	C_10_H_18_N_5_O_15_P_3_	0.0241 ± 0.0081	0.0213 ± 0.0069	0.0311 ± 0.0115*	0.0273 ± 0.0160
	29	2.2368	Psychosine sulfate^#^	HMDB0013046	C02744	C_24_H_47_NO_10_S	0.0584 ± 0.0251	0.0551 ± 0.0150	0.0797 ± 0.0336*	0.0641 ± 0.0144
	30	1.8309	Isopropyl‐diiodothyronine	HMDB0253678	—	C_18_H_19_I_2_NO_4_	0.0189 ± 0.0144*	0.0081 ± 0.0029	0.0125 ± 0.0036**	0.0081 ± 00022

*Note:* # indicates KEGG numbering. *indicates *p* < 0.05 and **indicates *p* < 0.01 compared with the model control group.

In the negative ion mode, eight potential biomarkers and five differential metabolites were identified from the 38 plasma metabolites identified. The differential metabolites were cytidine diphosphoglucose (CDP‐glucose), adenosine diphosphoglucose (ADP‐glucose), formamidopyrimidine nucleoside triphosphate, psychosine sulfate, and isopropyl‐diiodothyronine. Four of these (CDP‐glucose, ADP‐glucose, formamidopyrimidine nucleoside triphosphate, and psychosine sulfate) had a KEGG annotation numbering (Table 3). CDP‐glucose (*p* < 0.01) and isopropyl‐diiodothyronine (*p* < 0.05) levels were significantly lower in the model control group than in the sham‐operated group. Compared with the model control group, the nimodipine control group had significantly increased isopropyl‐diiodothyronine levels (*p* < 0.01), similar to the sham‐operated group. The levels of ADP‐glucose, formamidopyrimidine nucleoside triphosphate, and psychosine sulfate were significantly increased (*p* < 0.05).

The high and low levels of differential metabolites in each rat are illustrated in Figure 7D. PS(18:0/18:0) was highest in N‐2 (the second rat in the nimodipine control group) and lowest in K‐5. AppppA was highest in M‐1 and lowest in K‐5, and lysoPC (0:0/18:2(9Z,12Z)) was highest in Q‐3 and lowest in K‐6. The remaining differential metabolites were homologous. Accordingly, modeling and drug administration affected the metabolites in the normal rats in vivo.

The correlations between the differential metabolites are depicted in Figure 7D. lysoPC (0:0/18:0), docosanamide, lysoPC (0:0/18:2(9Z,12Z)), AppppA, and PS (18:0/18:0) were positively correlated. When lysoPC (0:0/18:0) levels increased, those of the other aforementioned metabolites increased accordingly, and the correlation coefficients decreased sequentially. lysoPC (0:0/18:0), CDP‐glucose, isopropyl‐diiodothyronine, lysoPC (18:1(11Z)/0:0), ADP‐glucose, PS (18:0/18:0), and formamidopyrimidine nucleoside triphosphate were negatively correlated. When lysoPC (0:0/18:0) levels increased, those of the other aforementioned metabolites decreased accordingly, and the correlation coefficients decreased sequentially.

#### 3.3.4 Metabolic Pathway Analysis

Among the 12 differential metabolites identified in the positive and negative ion modes, six had corresponding KEGG annotations. The six metabolites were imported into the MetaboAnalyst 5.0 (http: //www.metaboanalyst.ca/) platform for metabolic pathway analyses and were found to be involved in three metabolic pathways: formamidopyrimidine nucleoside triphosphate, in folate biosynthesis; lysoPC (18:1(11Z)/0:0), or 1‐acyl‐sn‐glycero‐3‐phosphocholine, in glycerophospholipid metabolism; and AppppA, in purine metabolism. The largest impact of differential metabolites was on folate biosynthesis (0.0972) (Table 4 and Figure 7E).

**TABLE 4:** Information on the three metabolic pathways.

Metabolic pathway	Participating metabolites	Raw *p*	−Log_10_(*p*)	Impact
Folate biosynthesis	Formamidopyrimidine nucleoside triphosphate	0.0528	1.2777	0.0972
Glycerophospholipid metabolism	LysoPC(18:1(11Z)/0:0)	0.0699	1.1554	0.0174
Purine metabolism	Diadenosine tetraphosphate	0.1256	0.9009	0

##### 3.3.4.1 Folate Biosynthesis and Cerebral Ischemia

Folate increases the signal expression of neurogenic locus notch homolog protein 1 (NOTCH1), hairy/enhancer of split (HES)1, and HES5 in the brain and the number of newborn hippocampal neurons and reduces cognitive function damage, thereby reducing the cerebral ischemia‐induced damage [99]. In addition, folate reduces the apoptosis rate in the brain tissue, decreases serum MDA levels, increases SOD and glutathione peroxidase (GSH‐PX) activities, participates in oxidative stress in the brain tissue, enhances antioxidant capacity, and increases the phosphorylation of extracellular signal‐regulated kinases 1/2 [100, 101]. Thus, the folate biosynthesis pathway (Figure 7b) may be an important mechanism for in vivo regulation of QSWZZP metabolism in cerebral ischemia. This is achieved by improving the antioxidant and cognitive capacities, decreasing apoptosis, and increasing the number of neurons. In the present study, formamidopyrimidine nucleoside triphosphate levels tended to increase in both the sham‐operated and QSWZZP groups compared with that in the model control group, suggesting that its production is facilitated by the folate biosynthesis pathway.

##### 3.3.4.2 Glycerophospholipid Metabolism and Cerebral Ischemia

Phospholipids are divided into two classes: glycerophospholipids and sphingolipids. Glycerophospholipids are the most abundant phospholipids, and phosphatidylcholine (PC) and phosphatidylethanolamine (PE) are the key intermediates of glycerophospholipid metabolism. Reportedly, after CI/RI, glycerophospholipid degradation in brain cell membranes significantly downregulated PC and PE levels in the rat cerebral cortex, ultimately exacerbating brain cell membrane damage [102, 103]. In the present study, the levels of lysoPC (18:1(11Z)/0:0) (i.e., 1‐acyl‐sn‐glycero‐3‐phosphocholine), a metabolite involved in glycerophospholipid metabolism, were significantly lower in the model control group than in the sham‐operated group. This indicated the inhibition of the glycerophospholipid metabolic pathway after cerebral ischemia, consistent with that reported in the literature. In contrast, lysoPC (18:1(11Z)/0:0) levels remained significantly lower in the QSWZZP group than in the model control group. This suggested that QSWZZP may not protect against cerebral ischemia via glycerophospholipid metabolic pathway modulation (Figure 7w).

##### 3.3.4.3 Purine Metabolism and Cerebral Ischemia

During infection, local ischemia–reperfusion and inflammation occur, often leading to cellular stress or death and the secretion of adenosine triphosphate (ATP), which is hydrolysed to produce adenosine. This activates the purinergic receptors, P1 and P2. P1 receptor signaling mitigates ischemia–reperfusion injuries, including that of the small bowel, transplanted organs, and the heart, and provides a protective effect [104]. In contrast, P2 receptor signaling promotes tissue damage and inflammation. Therefore, amelioration of ischemia–reperfusion injury requires selective activation or inhibition of purinergic receptor signaling. The levels of AppppA, a metabolite of the purine metabolic pathway, were significantly higher in the model control group than in the sham‐operated group. However, the protective effect of AppppA against cerebral ischemic injury has been reported [105], suggesting the activation of the purinergic receptors after AppppA release and stimulation of both P1 and P2 receptors. However, the P2 receptor was dominant. In contrast, AppppA levels did not change significantly in the QSWZZP group compared with those in the model control group. This suggested that QSWZZP administration did not interfere with AppppA release and that the purine metabolism pathway (Figure 7x) may not be the mechanism via which QSWZZP ameliorates cerebral ischemia.

### 3.4 P53/Cyt C/APAF‐1 Mitochondrial Apoptosis Pathway‐Based Treatment of Cerebral Ischemia

#### 3.4.1 Neurobehavioral Ratings and Cerebral Infarction Rates

The neurobehavioral grades are presented in Table 5. The normality test revealed that the data did not exhibit the characteristics of a normal/Gaussian distribution (*p* < 0.05). Hence, the non‐parametric Mann–Whitney test was performed. Compared with the sham‐operated group, the model control group exhibited significant neurobehavioral abnormalities at 2 h and 24 h after cerebral ischemia (*p* < 0.01). This manifested as abnormal extension of the left forelimb (paw), circling to the left, and tilting to the left. Compared with the model control group, the neurobehavioral abnormalities of the various drug‐administered groups at 2 h after cerebral ischemia did not exhibit any significant changes (*p* > 0.05). The neurobehavioral abnormalities at 24 h after cerebral ischemia in the nimodipine control, QSWZZP medium‐dose, and QSWZZP high‐dose groups were significantly improved (*p* < 0.01).

**FIGURE 8:**
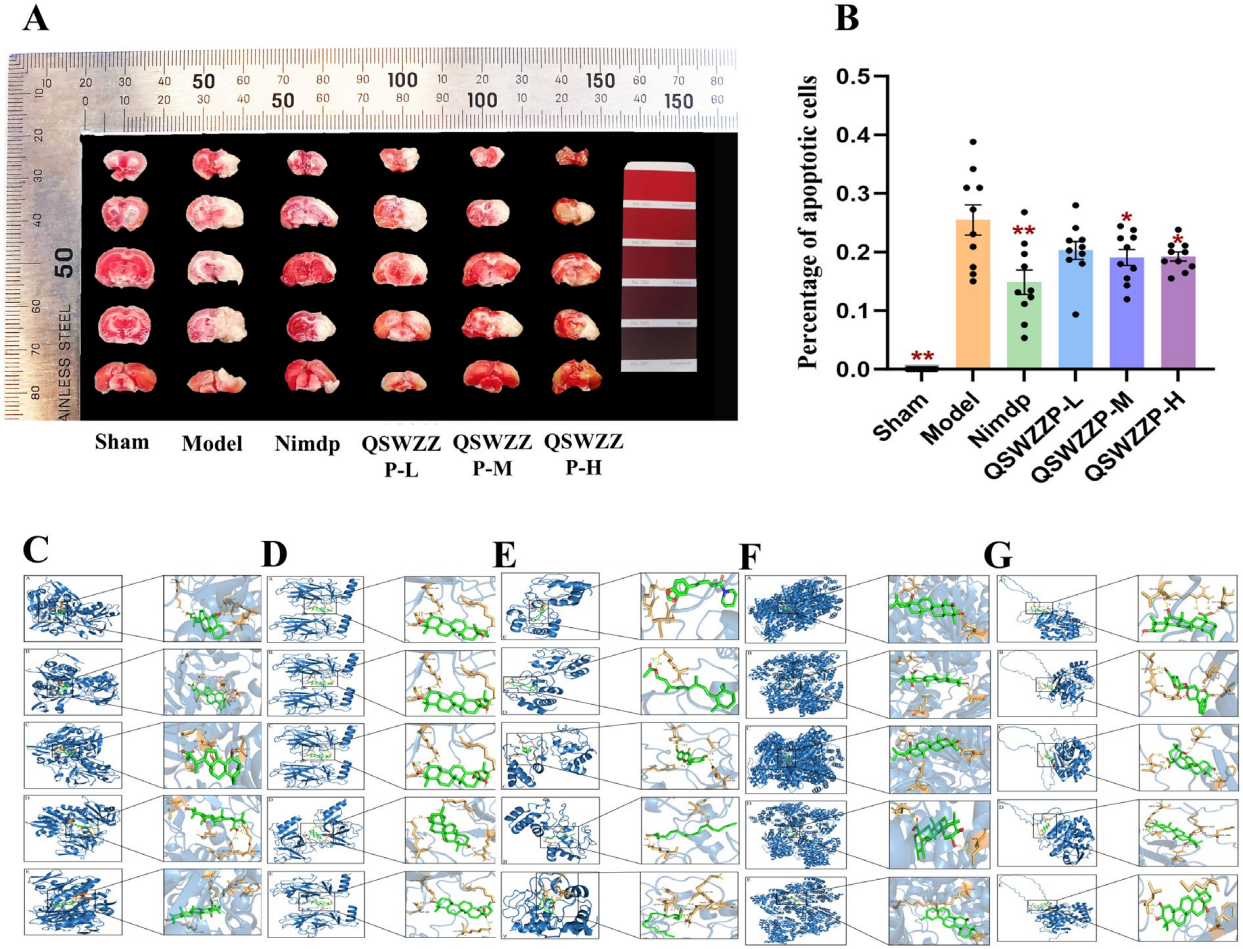
Effects of QSWZZP on the ratio of cerebral infarction in MCAO rats ((A) TTC staining. (B) Ratio of cerebral infarction). Visualization of the binding activities of the top‐five potential active ingredients docking with AIF, P53, Cyt C, APAF‐1, and NDRG4 (C) Cholic acid, porcine deoxycholic acid, ursodeoxycholic acid, Sumatran resinoic acid, and arjunic acid docked with AIF (D) corosolic acid, hawthornic acid, arjunic acid, Sumatran resinoic acid, and Thai resinoic acid docked with P53. (E) Peanut tetra enoic acid, (9Z,11E)‐13‐oxooctadeca‐9,11‐dienoic acid, 6‐methoxy‐2‐(2‐(3‐methoxyphenyl) ethyl) chromone, rosin oil acetate, and piperine were docked to Cyt C (F) hawthornic acid, Thai resinoic acid, arjunic acid, corosolic acid, and Sumatran resinoic acid were docked to APAF‐1. (G) Sumatran resinoic acid, amlaic acid, Thai resinoic acid, arjunic acid, and corosolic acid docked with NDRG4. *p < 0.05 versus model.

The cerebral infarction rates are presented in Table 5 and Figure 8A,B. The normality test revealed that the data exhibited the characteristics of a normal/Gaussian distribution (*p* > 0.05); hence, the independent samples underwent a t‐test. Compared with the sham‐operated group, the model control group had significantly increased cerebral infarction rates (*p* < 0.01), indicating the successful preparation of the cerebral ischemia model. Compared with the model control group, the nimodipine control group had significantly decreased cerebral infarction rates (*p* < 0.01), while the QSWZZP medium‐ and high‐dose groups had significantly decreased cerebral infarction rates (*p* < 0.05) as well.

**TABLE 5:** Effects of QSWZZP on neuroethology, apoptosis in hippocampal CA3 and diencephalon and the cerebral infarction rates in the MCAO model rats(*n* = 10, $\bar{x}\pm s$).

Groups	Dosage (mg·kg^−1^)	Neuroethology												Cerebral infarction rate (%)	Percentage of apoptotic cells in hippocampal CA3 region (%)	Percentage of apoptotic cells in diencephalon (%)
		2 h						24 h								
		−	+	++	+++	++++	*p*‐value	−	+	++	+++	++++	*p*‐value			
Sham operation group	—	10	0	0	0	0	0.002**	10	0	0	0	0	0.000**	0.00 ± 0.00**	0.00 ± 0.00	0.00 ± 0.00**
Model group	—	0	0	9	1	0	—	0	5	5	0	0	—	25.49 ± 8.19	2.66 ± 6.00	37.64 ± 23.23
Nimodipine control group	30.00	0	2	8	0	0	0.088	5	4	1	0	0	0.008**	14.85 ± 7.49**	4.83 ± 15.07	24.16 ± 16.61
QSWZZP low dose group	33.34	0	0	9	1	0	1.000	0	8	2	0	0	0.170	20.30 ± 4.81	0.06 ± 0.18	28.41 ± 29.68
QSWZZP medium dose group	66.68	0	0	9	1	0	1.000	5	5	0	0	0	0.002**	19.08 ± 4.31*	0.12 ± 0.27	23.69 ± 23.23
QSWZZP high dose group	133.36	0	2	8	0	0	0.088	6	4	0	0	0	0.001**	19.25 ± 2.47*	0.06 ± 0.20	18.70 ± 19.98*

*Note:* Compared with the model control group, *p < 0.05, * *p < 0.01.

#### 3.4.2 Histopathological Changes and Apoptotic Cells in the Ischemic Side of the Brain

##### 3.4.2.1 HE Staining

The results are presented in Table 6 and Figure 8A. The normality test revealed that the data did not exhibit the characteristics of a normal/Gaussian distribution (*p* < 0.05); hence, the non‐parametric Mann–Whitney test was performed. At 24 h after cerebral ischemia, compared with the sham‐operated group, the model control group exhibited significant neuronal necrosis and glial cell proliferation in the cortical area (*p* < 0.01), significant degeneration and necrosis of the pyramidal cells in the hippocampal CA3 area (*p* < 0.05), some—albeit not significant—necrosis in the hippocampal CA1 area, and significant degeneration of the diencephalic neurons and proliferation of glial cells (*p* < 0.01). Compared with the model control group, the nimodipine control and QSWZZP groups exhibited no significant changes in the cortical area, and the degeneration and necrosis of vertebral cells in the hippocampal CA3 area were significantly improved in the QSWZZP low‐ and QSWZZP high‐dose groups (*p* < 0.05). Mesencephalic neuronal necrosis in the QSWZZP medium‐ and QSWZZP high‐dose groups was significantly improved (*p* < 0.05), while mesencephalic glial cell proliferation was significantly reduced in the QSWZZP high‐dose group (*p* < 0.05).

**TABLE 6:** Effects of QSWZZP on the pathological changes of the cortex, hippocampus, and diencephalon in ischemic brain tissue of the MCAO rat models (*n* = 10, $\bar{x}\pm s$).

Groups	Dosage (mg/kg/d)	Cortical area												Hippocampus												Diencephalic region																							
		Cortical neuronal degeneration and necrosis						Cortical glial cell hyperplasia						Hippocampal ca1						Hippocampus CA3						Degeneration and necrosis of diencephalic neurons						Diencephalic glial cell proliferation						Diencephalon vascular congestion						Diencephalon hemorrhage					
		−	+	++	+++	++++	*p*	−	+	++	+++	++++	*p*	−	+	++	+++	++++	*p*	−	+	++	+++	++++	*p*	−	+	++	+++	++++	*p*	−	+	++	+++	++++	*p*	−	+	++	+++	++++	*p*	−	+	++	+++	++++	*p*
Sham operation group	—	10	0	0	0	0	0.0000**	10	0	0	0	0	0.0010**	10	0	0	0	0	0.3170	10	0	0	0	0	0.0300*	10	0	0	0	0	0.0000**	10	0	0	0	0	0.0010**	10	0	0	0	0	0.0670	10	0	0	0	0	0.1470
Model group	—	1	2	1	6	0	—	2	3	5	0	0	—	9	0	0	1	0	—	6	3	0	1	0	—	0	3	1	6	0	—	2	5	3	0	0	—	7	3	0	0	0	—	8	1	1	0	0	—
Nimodipine control group	30	1	1	0	8	0	0.4270	3	0	7	0	0	0.6350	8	1	0	1	0	0.5840	8	1	0	1	0	0.3990	1	4	2	3	0	0.1930	4	4	2	0	0	0.3720	8	2	0	0	0	0.6150	9	1	0	0	0	0.5030
QSWZZP low dose group	33.34	3	0	0	7	0	0.9640	3	0	7	0	0	0.6350	10	0	0	0	0	0.3170	10	0	0	0	0	0.0300*	0	4	2	4	0	0.4550	4	6	0	0	0	0.1090	9	1	0	0	0	0.2760	8	1	1	0	0	1.0000
QSWZZP medium dose group	66.68	1	1	0	8	0	0.4270	2	0	8	0	0	0.2840	8	2	0	0	0	0.6260	8	2	0	0	0	0.3000	1	6	2	1	0	0.0280*	5	5	0	0	0	0.0620	9	1	0	0	0	0.2760	7	3	0	0	0	0.7260
QSWZZP high dose group	133.36	3	1	0	6	0	0.6990	6	4	0	0	0	0.9330	9	0	0	1	0	1.0000	10	0	0	0	0	0.0300*	4	2	4	0	0	0.0100*	6	4	0	0	0	0.0320*	10	0	0	0	0	0.0670	10	0	0	0	0	0.1470

*Note:* No lesion is recorded as (—). A 4‐level method is used for lesions: mild (+), mild (+ +), moderate (+ + +), and severe (+ + + +). Compared with the model control group, *p < 0.05, **p < 0.01.

##### 3.4.2.2 TUNEL Staining

The results are presented in Table 5 and Figure 8B. The normality test revealed that the data did not exhibit the characteristics of normal/Gaussian distribution (*p* < 0.05). Hence, the non‐parametric Mann–Whitney test was performed. At 24 h of cerebral ischemia, the apoptosis rate in the hippocampal CA3 area in the model control group did not change significantly compared with that in the sham‐operated group (*p* > 0.05). However, it tended to increase, and the apoptosis rate in the mesencephalic area was significantly increased (*p* < 0.01). Compared with the model control group, the nimodipine control and QSWZZP groups did not exhibit significant changes in the apoptosis rate in the hippocampal CA3 area (*p* > 0.05). However, it was reduced by various degrees in the QSWZZP groups. Moreover, the apoptosis rate was significantly reduced in the QSWZZP high‐dose groups (*p* < 0.05), suggesting that QSWZZP could reduce apoptosis.

### 3.5 Screening of QSWZZP Chemical Constituents Against Mitochondria‐Mediated Apoptosis

#### 3.5.1 Potential Active Components and Target Proteins

Based on the aforementioned screening method of potential active components and target proteins, 33 potential active components were obtained. Twenty‐eight of these were formulated components, six were blood‐entry components (two were formulated ingredients as well), and one was a brain‐entry component. Table 7 presents the specific information.

**TABLE 7:** Information of QSWZZP's potential active components and its docking results with the target proteins.

Serial number	English name	GI	DL	Binding energy (kcal/mol)				
				AIF	P53	Cyt C	Apaf‐1	NDRG4
1	Gallic acid	High	3yes	−4.42	−5.54	−7.35	−4.14	−4.0
2	Apocynin	High	4yes	−5.5	−5.09	−6.64	−4.5	−4.74
3	Caffeic acid	High	4yes	−5.69	−5.51	−9.03	−4.6	−4.61
4	Ellagic acid	High	3yes	−8.22	−6.34	−6.94	−5.49	−5.64
5	(8R)‐evofolin B	High	5yes	−5.36	−5.32	−6.33	−3.88	−3.66
6	Agarotetrol	High	4yes	−5.96	−6.39	−7.41	−5.01	−4.95
7	3′‐hydroxy‐8‐methoxyvestitol	High	5yes	−7.31	−6.63	−6.01	−6.28	−5.3
8	Liquiritigenin	High	5yes	−8.17	−6.86	−8.7	−6.5	−5.77
9	Luteolin	High	5yes	−7.22	−6.48	−8.19	−5.53	−5.63
10	4′, 7‐Dimethoxyisoflavone	High	5yes	−7.8	−6.35	−8.38	−6.06	−5.9
11	Formononetin	High	5yes	−8.39	−6.33	−8.18	−6.14	−5.62
12	23‐Hydroxytormentic acid	High	4yes	−7.67	−8.34	−7.91	−6.58	−6.37
13	Arjungenin	High	4yes	−8.06	−7.89	−7.53	−6.66	−7.23
14	Hyocholic acid	High	5yes	−9.53	−7.85	−8.7	−6.92	−6.59
15	6‐Methoxy‐2‐[2‐(3‐methoxyphenyl) ethyl]chromone	High	5yes	−7.45	−6.28	−9.52	−6.13	−5.81
16	Ursodeoxycholic acid	High	5yes	−9.4	−8.3	−8.64	−7.11	−6.37
17	Piperine	High	5yes	−7.92	−6.98	−9.21	−6.84	−6.19
18	Arjunic acid	High	3yes	−8.93	−8.84	−8.21	−7.84	−6.92
19	Asiatic acid	High	3yes	−7.82	−8.18	−7.83	−6.63	−6.79
20	Hyodeoxycholic acid	High	5yes	−9.44	−8.1	−8.64	−7.38	−6.79
21	Chenodeoxycholic acid	High	5yes	−8.8	−8.15	−8.82	−6.61	−6.85
22	Sumaresinolic acid	High	2yes	−9.0	−8.8	−9.12	−7.5	−8.03
23	Maslinic acid	High	2yes	−8.79	−9.06	−8.41	−7.99	−6.92
24	Corosolic acid	High	2yes	−8.54	−9.18	−8.23	−7.83	−7.05
25	(9Z,11E)‐13‐oxooctadeca‐9,11‐dienoic acid	High	3yes	−5.43	−4.29	−9.97	−4.41	−3.26
26	Hemerocallal A	High	5yes	−8.32	−7.7	−8.66	−6.77	−6.1
27	Siaresinolic acid	High	2yes	−8.79	−8.58	−8.64	−7.85	−7.55
28	Glyasperin C	High	5yes	−7.6	−6.37	−8.34	−5.79	−5.67
29	Obtusin	High	5yes	−6.02	−6.37	−7.71	−6.01	−5.63
30	Nonadecylic acid	High	2yes	−4.54	−3.94	−8.52	−3.24	−3.6
31	Retinol acetate	High	2yes	−7.85	−6.99	−9.43	−6.24	−6.68
32	Arachidonic acid	High	5yes	−5.14	−4.93	−10.67	−3.17	−3.24
33	Phyllaemblic acid	High	4yes	−8.41	−7.68	−7.94	−6.47	−7.81

#### 3.5.2 Molecular Docking Results

The docking results are presented in Table 7. Binding energies ≤ −4.0, ≤ −5.0, and ≤ −7.0 kcal/mol indicate that a small molecule has some, favorable, and significant binding activity, respectively, with the protein [106, 107]. The results revealed that after the 33 potential active ingredients were bound to five proteins, 157 had binding energies ≤ −4.0 kcal/mol (95% of the total), 145 had binding energies ≤ −5.0 kcal/mol (88% of the total), and 79 had binding energies ≤ −7.0 kcal/mol (48% of the total).

Most of the components exhibited good protein binding activity. Hence, binding energy ≤ −7.0 kcal/mol was chosen as the screening index value, and the results of the top‐five absolute values of the binding energies of the potentially active components docking with AIF, P53, Cyt C, APAF‐1, and NDRD4 were visualized and presented using Pymol 2.1. Among the 33 potentially active components, 24 docked with AIF and had binding energies ≤ −7.0 kcal/mol (significant binding activity). Cholic acid, which formed hydrogen bonds with LYS‐342, GLU‐314, LYS‐177, and SER‐176, had the highest binding activity (binding energy: −9.53 kcal/mol), followed by porcine deoxycholic acid, ursodeoxycholic acid, Sumatran resin acid, and arjunic acid, with binding energies of −9.44, −9.4, −9.4, −9.0, −9.0, −9.0, and −9.0, 9.4, −9.0, and −8.93 kcal/mol, respectively. The docking results are presented in Figure 8C.

Fourteen components docked with P53 and had binding energies ≤ −7.0 kcal/mol (significant binding activity). The top five components were corosolic acid, hawthorn acid, arjunic acid, Sumatran resin acid, and Thai resin acid, with binding energies of −9.18, −9.06, −8.84, −8.8, and −8.58 kcal/mol, respectively, with corosolic acid forming hydrogen bonds with ASP‐186, ARG‐196, and LYS‐139. The docking results are presented in Figure 8D.

Five components docked with Cyt C and had binding energies ≤ −7.0 kcal/mol (significant binding activity). These components, in descending order of binding activity, were arachidonic acid, (9Z,11E)‐13‐oxooctadeca‐9,11‐dienoic acid, 6‐methoxy‐2‐(2‐(3‐methoxyphenyl)ethyl) chromone, rosin acetate, and piperine, with binding energies of −10.67, −9.97, −9.97, −9.43, −9.52, −9.97, −9.97, and −9.43 kcal/mol, respectively. Piperine had binding energies of −10.67, −9.97, −9.52, −9.43, and −9.21 kcal/mol. The docking results are presented in Figure 8E.

Among the 33 potentially active components, hawthorn acid had the best docking activity with APAF‐1, forming hydrogen bonds with ASN‐562 and ASP‐82, with a binding energy of −7.99 kcal/mol, followed by Thai resinous acid, arjunic acid, corosolic acid, and Sumatran resinous acid. The specific results are presented in Figure 8F.

Five components docked with NDRG4 and had binding energies≤ −7.0 kcal/mol. These five components, in descending order of binding activity, were Sumatran resinoic acid, arjugenin, Thai resinoic acid, arjunic acid, and corosolic acid, with binding energies of −8.03, −7.81, −7.55, −7.23, and −7.05 kcal/mol, respectively. The docking results are presented in Figure 8G.

### 3.6 mRNA Expression of Mitochondrial Apoptosis Pathway‐Related Factors in the Ischemic Brain Tissue

**FIGURE 9:**
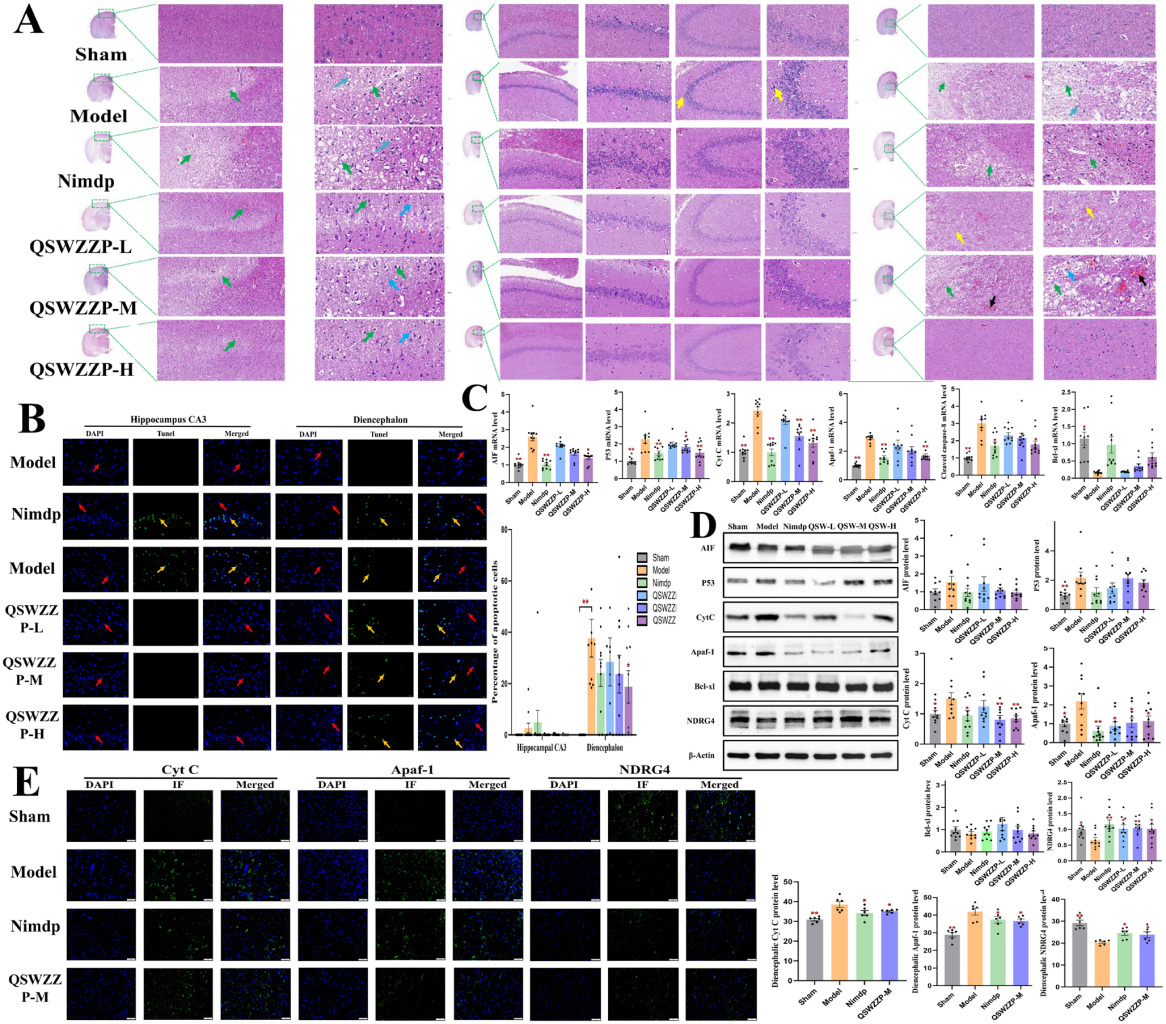
Effects of QSWZZP on HE staining, TUNEL staining, RT‐PCR, WB, and IF in cerebral ischemia (A) HE staining: (↑) indicates neuronal degeneration and necrosis, (↑) indicates glial cell proliferation, (↑) indicates vertebral cell degeneration and necrosis, (↑) indicates hemorrhage. (B) TUNEL staining and apoptotic cell ratio: (↑) indicates normal cells. (↑) indicates apoptotic cells. (C) PCR: MRNA expression of AIF, P53, Cyt C, APAF‐1, cleaved caspase‐8, and BCL‐XL. (D) WB: Protein expression of AIF, P53, Cyt C, APAF‐1, BCL‐XL, and NDRG4. (E) IF: Fluorescence expression of Cyt C, APAF‐1, and NDRG4 in the diencephalon. *p < 0.05 versus model.

The results are presented in Table 8 and Figure 9C. The normality test revealed that the data exhibited the characteristics of a normal/Gaussian distribution (*p* > 0.05). Hence, the independent samples underwent a t‐test. At 24 h after cerebral ischemia, compared with the sham‐operated group, the model control group exhibited significantly increased AIF, P53, Cyt C, APAF‐1, and cleaved caspase‐8 mRNA expressions (*p* < 0.01) and a significantly decreased BCL‐XL mRNA expression (*p* < 0.05). Compared with the model control group, the nimodipine control group exhibited significantly decreased AIF, P53, Cyt C, and APAF‐1 mRNA expressions (*p* < 0.01) and a significantly decreased cleaved caspase‐8 mRNA expression (*p* < 0.05). Furthermore, AIF, P53, Cyt C, and APAF‐1 mRNA expressions in the QSWZZP high‐dose group were significantly decreased (*p* < 0.01), and AIF and cleaved caspase‐8 mRNA expressions were significantly decreased (*p* < 0.05). Cyt C mRNA expression in the QSWZZP middle‐dose group was significantly decreased (*p* < 0.01), and P53 mRNA expression was significantly decreased (*p* < 0.05). In addition, AIF, P53, Cyt C, APAF‐1, and cleaved caspase‐8 mRNA expressions in the remaining groups were not statistically significant. Although no significant change in BCL‐XL mRNA expression was observed in all groups, it tended to increase with increasing QSWZZP dosage in each QSWZZP group.

**TABLE 8:** Effects of QSWZZP on the expression of mitochondrial apoptosis‐related mrna, proteins, and diencephalic cyt c, apaf‐1, and NDRG4 proteins in brain tissue of MCAO model rats ($\bar{x}\pm s$).

Groups	Dosages (mg/kg/d)	Related indicators	AIF	P53	Cyt C	Apaf‐1	Cleaved caspase‐8	Bcl‐xl	NDRG4
Sham operation group	—	mRNA(*n* = 10)	1.024 ± 0.245**	1.017 ± 0.204**	1.020 ± 0.223**	1.006 ± 0.122**	1.015 ± 0.194**	1.148 ± 0.593*	—
		Proteins(*n* = 10)	1.0000 ± 0.5099	1.0000 ± 0.4383**	1.0000 ± 0.3632*	1.0000 ± 0.4947*	—	1.0000 ± 0.4214	1.0000 ± 0.4035*
		Immunofluorescence of diencephalic regions(*n* = 6)	—	—	30.8793 ± 1.4912**	28.8688 ± 3.2155**	—	—	29.1987 ± 3.0478**
Model group	—	mRNA(*n* = 10)	2.604 ± 0.741	2.277 ± 0.714	2.424 ± 0.402	2.892 ± 0.270	3.006 ± 0.739	0.163 ± 0.038	—
		Proteins(*n* = 10)	1.5248 ± 1.0967	2.1738 ± 1.2022	1.5060 ± 0.6190	2.1897 ± 1.2963	—	0.8279 ± 0.2650	0.6634 ± 0.2576
		Immunofluorescence of diencephalic regions(*n* = 6)	—	—	38.5312 ± 3.6812	41.9547 ± 4.8808	—	—	20.3916 ± 1.0713
Nimodipine control group	30.00	mRNA(*n* = 10)	1.026 ± 0.253**	1.475 ± 0.407**	0.999 ± 0.362**	1.547 ± 0.405**	1.712 ± 0.651*	0.972 ± 0.756	—
		Proteins(*n* = 10)	0.9653 ± 0.7331	1.1965 ± 0.9152	0.9464 ± 0.5229*	0.6194 ± 0.8199**	—	0.9199 ± 0.3376	1.1688 ± 0.4167**
		Immunofluorescence of diencephalic regions(*n* = 6)	—	—	34.2637 ± 3.0909*	37.3174 ± 4.7537*	—	—	24.5529 ± 2.5582*
QSWZZP low dose group	33.34	mRNA(*n* = 10)	2.097 ± 0.306	2.003 ± 0.323	2.056 ± 0.340	2.421 ± 1.108	2.323 ± 0.448	0.188 ± 0.019	—
		Proteins(*n* = 10)	1.4439 ± 1.2788	1.4501 ± 1.1403	1.2448 ± 0.6263	0.8992 ± 0.6355*	—	1.2564 ± 0.9306	1.0277 ± 0.3849*
QSWZZP medium dose group	66.68	mRNA(*n* = 10)	1.639 ± 0.322	1.843 ± 0.397*	1.553 ± 0.431**	2.053 ± 0.834	2.155 ± 0.873	0.357 ± 0.193	—
		Proteins(*n* = 10)	1.0651 ± 0.4921	2.1359 ± 0.9281	0.8098 ± 0.4299**	1.0480 ± 0.8227*	—	0.9805 ± 0.5508	1.0441 ± 0.3011**
		Immunofluorescence of diencephalic regions(*n* = 6)	—	—	34.2637 ± 3.0909*	36.1549 ± 2.5066*	—	—	23.8108 ± 3.3002*
QSWZZP high dose group	133.36	mRNA(*n* = 10)	1.387 ± 0.286*	1.504 ± 0.540**	1.322 ± 0.453**	1.676 ± 0.393**	1.767 ± 0.678*	0.620 ± 0.369	—
		Proteins(*n* = 10)	0.9542 ± 0.3704	1.8332 ± 0.6518	0.8459 ± 0.2955**	1.1324 ± 0.8854*	—	0.8361 ± 0.3344	1.0192 ± 0.4471*

*Note:* Compared with the model control group, *p < 0.05, **p < 0.01.

### 3.7 Protein Expression of Mitochondrial Apoptosis Pathway‐Related Factors in the Ischemic Brain Tissue

The results are presented in Table 8 and Figure 9D. The normality test revealed that the data exhibited the characteristics of normal/Gaussian distribution (*p* > 0.05). Hence, the independent samples underwent a t‐test. At 24 h after cerebral ischemia, compared with the sham‐operated group, the model control group exhibited significant increased P53 (*p* < 0.01), Cyt C and APAF‐1 (*p* < 0.05), and NDRG4 (*p* < 0.05) protein expressions. Although AIF and BCL‐XL protein expressions did not change significantly, they tended to increase or decrease (*p* > 0.05). Compared with the model control group, the nimodipine control group exhibited a significantly decreased APAF‐1 protein expression (*p* < 0.01), a significantly increased NDRG4 protein expression (*p* < 0.01), and a significantly decreased Cyt C protein expression (*p* < 0.05). Cyt C protein expression in the QSWZZP high‐dose group was significantly decreased (*p* < 0.01), and APAF‐1 protein expression was significantly decreased (*p* < 0.05) as well, while NDRG4 protein expression was significantly increased (*p* < 0.05). Cyt C protein expression in the QSWZZP middle‐dose group was significantly decreased (*p* < 0.01), while NDRG4 protein expression was significantly increased (*p* < 0.01), and APAF‐1 protein expression was significantly decreased (*p* < 0.05). In the QSWZZP low‐dose group, APAF‐1 protein expression was significantly decreased (*p* < 0.05) and NDRG4 protein expression was significantly increased (*p* < 0.05). Although no significant difference was observed in AIF, P53, and BCL‐XL protein expression in each group, all decreased or increased to varying degrees (*p* > 0.05).

### 3.8 Protein Expression of Cyt C, APAF‐1, and NDRG4 in the Mesencephalic Region of the Ischemic Brain Tissue

The results are presented in Table 8 and Figure 9E. The normality test revealed that the data exhibited the characteristics of a normal/Gaussian distribution (*p* > 0.05). Hence, the one‐way ANOVA test was performed. The fluorescence intensity of Cyt C and APAF‐1 in the mesencephalic area in the model control group was significantly higher than that in the sham‐operated group (*p* < 0.01), while the fluorescence intensity of NDRG4 was significantly lower (*p* < 0.01). Compared with the model control group, the nimodipine control and the QSWZZP medium‐dose groups exhibited significantly decreased fluorescence intensity of Cyt C and APAF‐1 in the mesencephalic area (*p* < 0.05), while the fluorescence intensity of NDRG4 was significantly increased (*p* < 0.05).

## 4 Discussion

Most of the qualitative studies on QSWZZP chemical composition have focused on its mineral components, with fewer studies on botanicals and animal‐origin components. Regarding in vitro characterization, Wu et al.'s LIBS‐based qualitative study found more than 10 elements [26]. Suo et al. used inductively coupled plasma (ICP)‐atomic emission spectroscopy to explore > 20 elements of QSWZZP, most of which were constant elements, such as potassium, sodium, magnesium, calcium, phosphorus, and a small amount of iron, copper, aluminum, zinc, gold, and other trace elements. The content of heavy metal elements, such as mercury and lead, after digestion and determination in artificial gastric juice was found to be considerably lower than the concentration of the original liquid, beyond the dose capable of causing damage in humans [108]. Han et al. used a HPLC‐tunable ultra violet‐evaporative light scattering detector to qualitatively analyze hyodeoxycholic acid and deoxycholic acid in QSWZZP [109]. Xu et al. applied HPLC to qualitatively analyze three components of chickpea budin A, piperine, and glycyrrhizin [7]. Xu used UPLC‐Q‐TOF‐MS to characterize 42 QSWZZP components, including coriolagin, agarotetrol, and ellagic acid, and clarified the cleavage pattern of each compound, thus providing a methodological basis for the qualitative analysis of QSWZZP [30]. In the present study, 33 new QSWZZP compounds were identified for the first time, which may serve as a foundational basis for subsequent serum pharmacochemical studies.

Regarding in vivo characterization, Li et al.'s study on the long‐term accumulation and distribution of QSWZZP's mineral components in animal tissues and organs [27], found that most of these mineral elements did not accumulate in animal tissues and organs and that these elements were mainly metabolized by the liver and kidneys and subsequently excreted, further illustrating the safety of QSWZZP. Moreover, Song et al. conducted an in vivo analysis of 18 QSWZZP elements (lithium, beryllium, scandium, vanadium, chromium, manganese, cobalt, nickel, copper, arsenic, strontium, silver, cadmium, cesium, barium, lead, gold, and mercury) using ICP‐MS and found that these elements were non‐toxic and mainly excreted through feces and that their accumulation in the blood, brain, liver, and kidney was extremely low. Furthermore, the changes produced by lithium, chromium, and cadmium in the brain were significant. hence, they hypothesized that this may be a material basis for QSWZZP's mechanism for cerebral ischemia treatment [29]. In the present study, UPLC‐Q‐TOF‐MS was used to further study the distribution of QSWZZP components in the blood and tissues of cerebral ischemia model rats. Fifteen components were identified in the blood, twenty‐one in the urine, three in the brain, seven in the liver, and four in the kidneys. Syringin reduces the expression of inflammatory factors(interleukin (IL)‐1β, tumor necrosis factor‐α, and IL‐6), and neutrophil infiltration and increases forkhead box O3A phosphorylation, thereby inhibiting nuclear factor kappa B expression and protecting against cerebral ischemia [110]. Liquiritigenin reportedly enters the ischemic rat brain tissue through the BBB, indicating that liquiritigenin may be an active compound in cerebral ischemia treatment [111]. Glycyrrhizin reduces cerebral infarction volume and attenuates CI/RI by lowering MDA levels, decreasing the number of positive apoptotic cells, and up‐regulating SOD, catalase, and GSH‐PX activities, indicating that glycyrrhizin may be a potential anti‐ischemic drug [112]. Bile acid components, such as ursodeoxycholic acid, protect against CI/RI by improving oxidative stress levels in the brain and modulating the protein kinase B signaling pathway [113]. In summary, syringin, liquiritigenin, glycyrrhizin, and ursodeoxycholic acid may be the active components of QSWZZP in cerebral ischemia treatment.

Regarding metabolomic studies, Liang investigated the endogenous metabolites of QSWZZP in cerebral ischemia model rats using gas chromatography (GC)‐MS and found that the metabolism mechanism was primarily related to the oxidative stress response and lipid, fatty acid, and energy metabolisms [114]. However, in the present study, owing to the limitations of the GC–MS technique, the UPLC‐Q‐TOF‐MS, which allows for high sensitivity and resolution, was chosen. Three metabolic pathways were obtained using data processing and analysis, and among the differential metabolites with an elevated trend in the drug‐administered group, only formamidopyrimidine nucleoside triphosphate was involved in the regulation of the relevant metabolic pathway.

Folate promotes the normal development and growth of the nervous system [100, 101], and the folate biosynthetic pathway may be the only pathway to regulate cerebral ischemia amelioration via QSWZZP. Folate increases signal expression of NOTCH1, HES1, and HES5, increases the number of newborn hippocampal neurons, decreases apoptosis rates in the brain tissues, decreases serum MDA levels, increases SOD and GSH‐PX activities, and enhances antioxidant capacity, alleviating CI/RI [99, 100, 101]. Moreover, glycerophospholipid and purinergic metabolisms are involved in the regulation of cerebral ischemia, with PC and PE metabolisms being the key intermediates. Cerebral ischemia leads to a significant decrease in both PC and PE levels, exacerbating the degree of damage to the brain cell membrane [102, 103]. However, purinergic metabolism ameliorates CI/RI by modulating P1 and P2 receptors, with P1 receptors attenuating the injury and P2 receptors promoting tissue damage and inflammation. Hence, selective activation or inhibition of purinergic receptor signaling is essential [105].

Apoptosis is a fundamental mechanism for biological processes such as embryonic development, tissue homeostasis, and immune defense. It occurs through highly regulated endogenous and exogenous pathways, with the mitochondria playing a key role in this process [115]. Available evidence indicates that structural and functional abnormalities of mitochondria may significantly exacerbate neural damage caused by cerebral ischemia–reperfusion injury [116]. One manifestation of this exacerbation is the activation of the mitochondria‐dependent apoptosis pathway, wherein ischemia–reperfusion injury significantly upregulates Cleaved Caspase‐3 and Bax—two critical proteins responsible for initiating the apoptotic cascade in mitochondria‐dependent neurons. Several factors can lead to mitochondrial dysfunction, such as accumulation and damage of reactive oxygen species, excess calcium ions, BBB disruption, abnormal opening of the mitochondrial permeability transition pore, abnormal mitochondrial membrane potential, mitochondrial DNA damage, and mitochondrial autophagy [117]. CI/RI triggers apoptosis, in which the mitochondrial apoptosis pathway dominates, mainly via the opening of the permeability transition pore to Cyt C from mitochondria into the cytoplasm, thereby activating the caspase‐3, ‐6, and ‐7 cascade response to exacerbate CI/RI [118]. In the present study, we investigated the mechanism by which QSWZZP alleviates cerebral ischemia via the mitochondrial apoptosis pathway. Compared with that in the model control group, AIF, P53, Cyt C, and APAF‐1 mRNA expressions in the QSWZZP groups were decreased, while BCL‐XL mRNA expression tended to increase. Moreover, QSWZZP effectively decreased Cyt C and APAF‐1 protein expressions and increased NDRG4 protein expression. Thus, QSWZZP mainly treats cerebral ischemia by modulating the P53/Cyt C/APAF‐1‐mediated mitochondrial apoptosis pathway.

**FIGURE 10:**
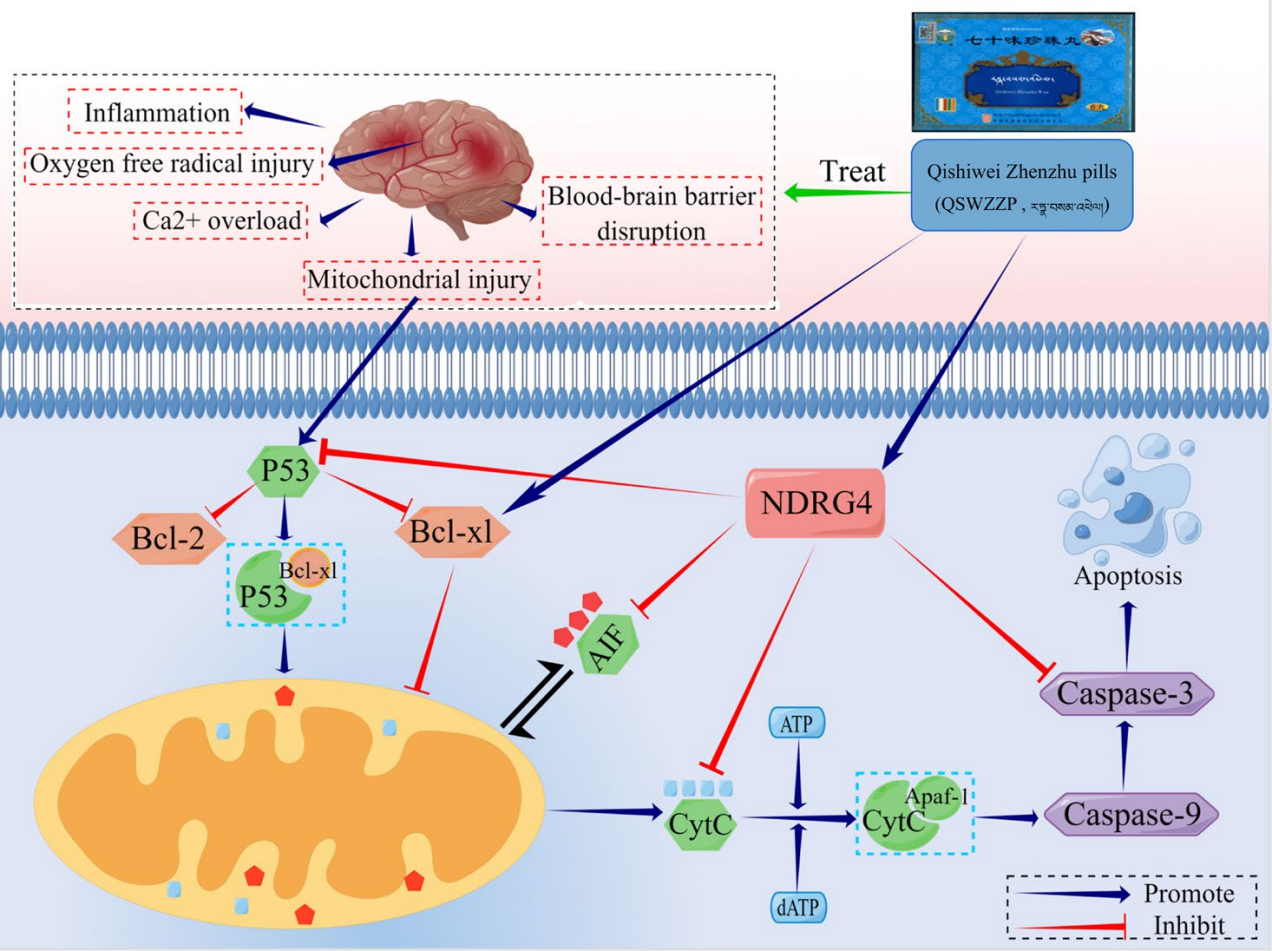
QSWZZP regulates mitochondria‐mediated apoptosis mechanism (Blue arrows indicate promotion, red lines indicate inhibition. this figure was drawn on the Figdraw platform, Figdraw ID: OAAIWac1el).

AIF is an initiating factor and a direct effector between the inner and outer mitochondrial membranes during apoptosis. When cells are stimulated, the permeability of the mitochondrial outer membrane changes, and AIF is released into the cytoplasm or transferred to the nucleus. Membrane permeability change and AIF release form a cyclic feedback to accelerate apoptosis [119]. AIF is regulated by *P53* as well [120], a recognized pro‐apoptotic gene with mutant and wild‐type protein variants. Wild‐type P53 is associated with CI/RI, and it upregulates the pro‐apoptotic *BAX* and down‐regulates the anti‐apoptotic *BCL‐2* and *BCL‐XL* to exert pro‐apoptotic functions [121]. Zou et al.'s investigation on the effect of miR‐130b‐regulated P53 on neuronal apoptosis in rats with focal cerebral ischemia, found that miR‐130b protects against CI/RI via P53 inhibition [122]. Cyt C, a cytochromeoxidase that plays a key role in mitochondria‐regulated respiration, is present in the outer and inner mitochondrial membranes, and it is a key apoptosis factor as well. During apoptosis, Cyt C is released into the cytoplasm and binds to APAF‐1, via ATP or dATP facilitation, and mainly activates the upstream caspase‐9 apoptosis initiator and the downstream caspase‐3 apoptosis effector [119]. Moreover, Cyt C promotes apoptosis by modulating the pro‐ and anti‐apoptotic *BAX* and anti‐apoptotic *BCL‐2* and *BCL‐XL* expressions. *APAF‐1* is an oncogene involved in the formation of an apoptosome complex and is located in the cytoplasm [123]. Cui et al. investigated the effects of APAF‐1, caspase‐9, and caspase‐3 using the Chinese herbal medicinal formula of promoting blood circulation and removing blood stasis [124]. They found that promoting blood circulation and removing blood stasis reduced APAF‐1, caspase‐9, and caspase‐3 in cerebral ischemia model rats, and inhibited the apoptosis of the hippocampal neurons by modulating the APAF‐1‐mediated caspase pathway. *BCL‐XL*, an anti‐apoptotic gene of the BCL family [125], inhibits the opening of the mitochondrial permeability transition pore, thereby inhibiting the release of Cyt C. NDRG4 reduces the protein expression of pro‐apoptosis‐related factors P53, BAX, Cyt C, and, caspase‐3, and exerts a neuroprotective effect [40, 126]. In summary, cerebral ischemia induces the initiation of P53 [127], which directly controls apoptosis by regulating the gene expression of *BAX*, *BCL‐2*, and *BCL‐XL*, or stimulates the opening of the mitochondrial membrane permeability transition pore, thereby releasing AIF and Cyt C into the periplasm and cytoplasm. This activates upstream caspase‐9 and downstream caspase‐3, triggering apoptosis, thus aggravating CI/RI. *BCL‐XL* indirectly participates in cerebral ischemia regulation. *NDRG4* significantly decreases the expression of apoptosis‐inducing factor proteins, such as P53, Cyt C, caspase‐3, and protects against CI/RI damage. The mechanism of action is presented in Figure 10.

Molecular docking is commonly used to screen the active ingredients of treatment drugs and predict the mechanism of drug action, which can improve experimental efficiency and reduce research blindness [128]. The docking components were divided into blood and brain components, such as cholic acid, ajmalic acid, eugenic acid, cassia, and arachidonic acid, and demonstrated significant binding energy with five target proteins. This confirmed that these blood and brain components were the active ingredients that played an anti‐apoptosis role in the mitochondria. The bile acid component, ursodeoxycholic acid, alleviates hepatic fibrosis by downregulating the protein expression of mitochondrial NIX [129]. Arachidonic acid can be derived in vivo from eicosanoids, including prostaglandin (PG) synthases, leukotriene synthases, thromboxanes, hydroxyeicosatetraenoic acid synthases, and epoxyeicosatetraenoic acid synthases, which play an important role in several physiological and pathophysiological processes, in particular, in mitochondrial apoptosis [115]. Notably, PGE2 exhibits an anti‐apoptotic effect on cancer cells and a pro‐apoptotic effect on most non‐cancer cells, such as fibroblasts and human lymphocytes. Intracellular and extracellular PGE2 exhibits opposite effects: pro‐apoptotic in intracellular environments and anti‐apoptotic in extracellular environments. Therefore, the levels of intracellular and extracellular PGE2 need to be balanced. PGD2 and its metabolite, 15d‐PGJ2, have pro‐apoptotic effects, and the mitochondrial proteins are the main targets of 15d‐PGJ2 in apoptotic cell death [115]. In summary, arachidonic acid derivatives of eicosanoids can be pro‐apoptotic or anti‐apoptotic by modulating the mitochondrial pathway, thus achieving specific therapeutic effects.

To the best of our knowledge, this is the first UPLC‐Q‐TOF‐MS‐based qualitative study investigating the blood‐enter and tissue‐distributed components of QSWZZP, used to treat cerebral ischemia. This study may help in determining the material basis of the drug's efficacy and its pharmacokinetics in future investigations. Other techniques, such as radioisotope labeling and nuclear magnetic resonance spectroscopy, can be combined to effectively differentiate between metabolic prototypes, metabolites, and complex matrices [66]. For example, Jaganath et al. investigated the role of intestinal flora in rutin metabolism using radioisotope labelling [130]. Quercetin was labeled with 14C, and quercetin metabolites, such as monooxygenates, dioxygenates, and methylene quinone intermediates, were obtained using HPLC‐RC‐MS/MS. The present study is the first to investigate the mechanism by which QSWZZP regulated the mitochondrial pathway for cerebral ischemia treatment, which was verified using RT‐PCR, WB, and IF. We found that QSWZZP downregulated pro‐apoptotic genes, such as *Cyt C* and *APAF‐1*, and up‐regulated anti‐apoptotic genes, such as *NDRG4*. Molecular docking identified Cyt C as an effective target of QSWZZP for cerebral ischemia treatment via the mitochondrial apoptosis pathway and clarified the active ingredient that inhibited mitochondrial apoptosis. Thus, QSWZZP can be used as a treatment option for cerebral ischemia.

Our preliminary research has thoroughly explored the clinical effectiveness and mechanism of action of QSWZZP and verified its effectiveness and safety yet again. However, challenges in the current research on QSWZZP remain. As a complex compound preparation in traditional medicine, QSWZZP's effectiveness is a primary concern, and the elucidation of the mechanism of action is a gradual process that necessitates a sustained period of time. Our study may assist in the safe and effective application of QSWZZP and provide a research route for the modernization of QSWZZP and similar complex Tibetan medicine prescriptions. Furthermore, this study primarily investigated the anti‐mitochondrial apoptosis effects of QSWZZP, while the impacts of QSWZZP on other phenotypes such as autophagy [131] and neuroplasticity warrant further exploration. Building on the findings of the present study, we plan to conduct more in‐depth studies that will further elucidate the mechanisms underpinning our current findings and expand their applicability in the future.

## 5 Conclusions

In this study, 33 new compounds were identified and their chemical compositions characterized in preliminary experiments and via a compound database. Thirteen were triterpenoids, nine were flavonoids, two were anthraquinones, two were chromones, and seven were other components. The distribution of QSWZZP components in the blood and tissues of MCAO rats was further investigated using UPLC‐Q‐TOF‐MS. Fifteen blood‐entry, 21 urine‐entry, 3 brain‐entry, 7 liver‐entry, and 4 kidney‐entry compounds were identified. Eleven of these components were prototypes identified both in previous studies and in this study. Six were non‐prototype metabolites and twenty‐eight were metabolites, which were mainly phase I metabolism derivatives, in which the prototype components underwent oxidation, reduction, hydrolysis, hydroxylation, deglycosylation, and demethylation, and phase II metabolism derivatives, in which they underwent acetylation and methylation. Eighty‐two plasma metabolites were identified in the positive and negative ion modes, 18 of which were potential biomarkers with VIP > 1. Twelve differential metabolites and three metabolic pathways were identified. Among these, the differential metabolite, formamidopyrimidine nucleoside triphosphate, was involved in the folate biosynthesis pathway, the only possible pathway that regulated cerebral ischemia amelioration via QSWZZP. The mRNA and protein expression of mitochondria‐related apoptotic factors AIF, P53, Cyt C, APAF‐1, cleaved caspase‐8, BCL‐XL, and NDRG4 were verified using RT‐PCR, WB, and IF, and the results suggested that QSWZZP treated cerebral ischemia primarily by regulating the P53/Cyt C/APAF‐1‐mediated mitochondrial apoptosis pathway. Molecular docking was used to screen QSWZZP's active ingredients that mediated mitochondrial apoptosis. Thirty‐three components docked with five mitochondrial apoptosis‐related target proteins, namely, AIF, P53, Cyt C, APAF‐1, and NDRG4, and 165docking results were obtained. Most of the active components (*n* = 145) were well bound to the target proteins, suggesting that QSWZZP likely treated cerebral ischemia by modulating the mitochondrial apoptosis pathway. Thus, this study may provide a reference for the material basis of anti‐apoptosis activity and mechanism of action of QSWZZP and a direction for future studies on QSWZZP efficacy and activity.

## Author Contributions

**Yinglian Song:** data curation, formal analysis, methodology, software, visualization, writing – original draft. **Guili Song:** data curation, validation, writing – review and editing. **Lame Lizhen:** validation, writing – review and editing. **Yan Liang:** data curation, formal analysis. **Mengtian Han:** investigation, methodology. **Yichu Yang:** methodology, validation. **Qiaoqiao Feng:** methodology, validation. **Yi Li:** methodology, validation. **Jingwen Zhang:** conceptualization, investigation. **Min Xu:** methodology, validation. **Yongzhong Zeweng:** supervision, validation. **Miao Jiang:** methodology, supervision, validation. **Zhang Wang:** conceptualization, methodology, supervision, validation.

## Ethics Statement

The experimental protocol was approved by the Animal Ethics Committee of Chengdu University of Traditional Chinese Medicine (Experimental animal welfare ethics number: 2020–39. Date for approval: 22 May 2023).

## Conflicts of Interest

The authors declare no conflicts of interest.

## Supporting information


Data S1.

## Data Availability

All data used during the study are available from the corresponding author upon reasonable request.
