# Supplementary figures and images for: Qishiwei Zhenzhu Pills Protect Against Cerebral Ischemia via the P53/Cytochrome C/Apoptotic Protease Activating Factor 1‐Mediated Mitochondrial Apoptosis Pathway
Source: CNS Neurosci Ther. 2025 Jun 26;31(6):e70476. doi: 10.1111/cns.70476 (PMC12198660; doi:10.1111/cns.70476)

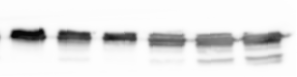

Supplement: Supplementary file 1 — Data S1. [file CNS-31-e70476-s001.zip › Supplemental Files/WB Figures/Full unedited gelblot for Figure 9d-AIF/1 Full unedited gelblot for Figure 9d-AIF.png]

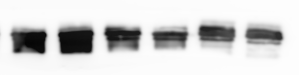

Supplement: Supplementary file 1 — Data S1. [file CNS-31-e70476-s001.zip › Supplemental Files/WB Figures/Full unedited gelblot for Figure 9d-AIF/2 AIF.tif]

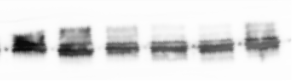

Supplement: Supplementary file 1 — Data S1. [file CNS-31-e70476-s001.zip › Supplemental Files/WB Figures/Full unedited gelblot for Figure 9d-AIF/3 AIF.tif]

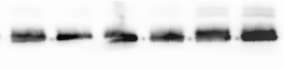

Supplement: Supplementary file 1 — Data S1. [file CNS-31-e70476-s001.zip › Supplemental Files/WB Figures/Full unedited gelblot for Figure 9d-AIF/4 AIF.tif]

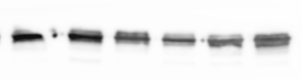

Supplement: Supplementary file 1 — Data S1. [file CNS-31-e70476-s001.zip › Supplemental Files/WB Figures/Full unedited gelblot for Figure 9d-AIF/5 AIF.tif]

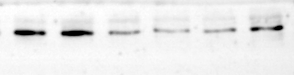

Supplement: Supplementary file 1 — Data S1. [file CNS-31-e70476-s001.zip › Supplemental Files/WB Figures/Full unedited gelblot for Figure 9d-Apaf-1/1 Full unedited gelblot for Figure 9d-Apaf-1.tif]

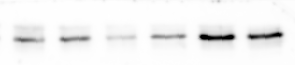

Supplement: Supplementary file 1 — Data S1. [file CNS-31-e70476-s001.zip › Supplemental Files/WB Figures/Full unedited gelblot for Figure 9d-Apaf-1/2 Apaf-1.tif]

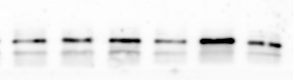

Supplement: Supplementary file 1 — Data S1. [file CNS-31-e70476-s001.zip › Supplemental Files/WB Figures/Full unedited gelblot for Figure 9d-Apaf-1/3 Apaf-1.tif]

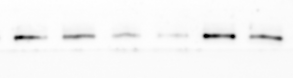

Supplement: Supplementary file 1 — Data S1. [file CNS-31-e70476-s001.zip › Supplemental Files/WB Figures/Full unedited gelblot for Figure 9d-Apaf-1/4 Apaf-1.tif]

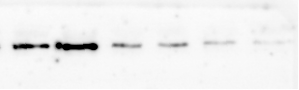

Supplement: Supplementary file 1 — Data S1. [file CNS-31-e70476-s001.zip › Supplemental Files/WB Figures/Full unedited gelblot for Figure 9d-Apaf-1/5 Apaf-1.tif]

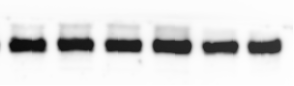

Supplement: Supplementary file 1 — Data S1. [file CNS-31-e70476-s001.zip › Supplemental Files/WB Figures/Full unedited gelblot for Figure 9d-Bcl-XL/1 Full unedited gelblot for Figure 9d-Bcl-XL.tif]

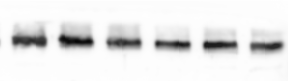

Supplement: Supplementary file 1 — Data S1. [file CNS-31-e70476-s001.zip › Supplemental Files/WB Figures/Full unedited gelblot for Figure 9d-Bcl-XL/2 Bcl-XL.tif]

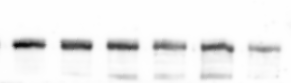

Supplement: Supplementary file 1 — Data S1. [file CNS-31-e70476-s001.zip › Supplemental Files/WB Figures/Full unedited gelblot for Figure 9d-Bcl-XL/3 Bcl-XL.tif]

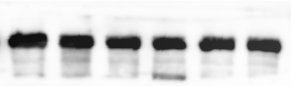

Supplement: Supplementary file 1 — Data S1. [file CNS-31-e70476-s001.zip › Supplemental Files/WB Figures/Full unedited gelblot for Figure 9d-Bcl-XL/4 Bcl-XL.tif]

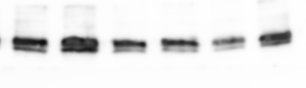

Supplement: Supplementary file 1 — Data S1. [file CNS-31-e70476-s001.zip › Supplemental Files/WB Figures/Full unedited gelblot for Figure 9d-Bcl-XL/5 Bcl-XL.tif]

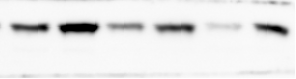

Supplement: Supplementary file 1 — Data S1. [file CNS-31-e70476-s001.zip › Supplemental Files/WB Figures/Full unedited gelblot for Figure 9d-Cytc/1 Full unedited gelblot for Figure 9d-Cytc.tif]

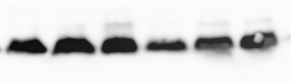

Supplement: Supplementary file 1 — Data S1. [file CNS-31-e70476-s001.zip › Supplemental Files/WB Figures/Full unedited gelblot for Figure 9d-Cytc/2 Cytc.tif]

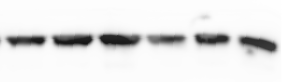

Supplement: Supplementary file 1 — Data S1. [file CNS-31-e70476-s001.zip › Supplemental Files/WB Figures/Full unedited gelblot for Figure 9d-Cytc/3 Cytc.tif]

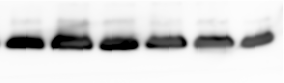

Supplement: Supplementary file 1 — Data S1. [file CNS-31-e70476-s001.zip › Supplemental Files/WB Figures/Full unedited gelblot for Figure 9d-Cytc/4 Cytc.tif]

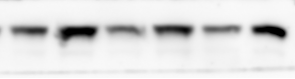

Supplement: Supplementary file 1 — Data S1. [file CNS-31-e70476-s001.zip › Supplemental Files/WB Figures/Full unedited gelblot for Figure 9d-Cytc/5 Cytc.tif]

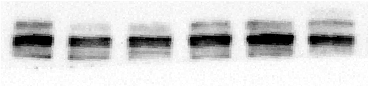

Supplement: Supplementary file 1 — Data S1. [file CNS-31-e70476-s001.zip › Supplemental Files/WB Figures/Full unedited gelblot for Figure 9d-NDRG4/1 Full unedited gelblot for Figure 9d-NDRG4.tif]

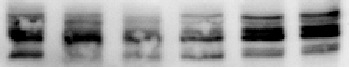

Supplement: Supplementary file 1 — Data S1. [file CNS-31-e70476-s001.zip › Supplemental Files/WB Figures/Full unedited gelblot for Figure 9d-NDRG4/2 NDRG4.tif]

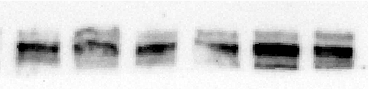

Supplement: Supplementary file 1 — Data S1. [file CNS-31-e70476-s001.zip › Supplemental Files/WB Figures/Full unedited gelblot for Figure 9d-NDRG4/3 NDRG4.tif]

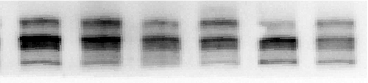

Supplement: Supplementary file 1 — Data S1. [file CNS-31-e70476-s001.zip › Supplemental Files/WB Figures/Full unedited gelblot for Figure 9d-NDRG4/4 NDRG4.tif]

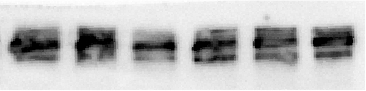

Supplement: Supplementary file 1 — Data S1. [file CNS-31-e70476-s001.zip › Supplemental Files/WB Figures/Full unedited gelblot for Figure 9d-NDRG4/5 NDRG4.tif]

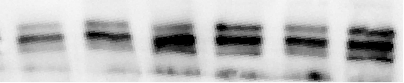

Supplement: Supplementary file 1 — Data S1. [file CNS-31-e70476-s001.zip › Supplemental Files/WB Figures/Full unedited gelblot for Figure 9d-NDRG4/6 NDRG4.tif]

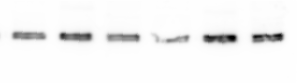

Supplement: Supplementary file 1 — Data S1. [file CNS-31-e70476-s001.zip › Supplemental Files/WB Figures/Full unedited gelblot for Figure 9d-P53/1 Full unedited gelblot for Figure 9d-P53.tif]

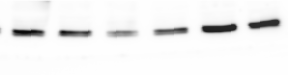

Supplement: Supplementary file 1 — Data S1. [file CNS-31-e70476-s001.zip › Supplemental Files/WB Figures/Full unedited gelblot for Figure 9d-P53/2 P53.tif]

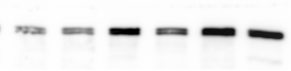

Supplement: Supplementary file 1 — Data S1. [file CNS-31-e70476-s001.zip › Supplemental Files/WB Figures/Full unedited gelblot for Figure 9d-P53/3 P53.tif]

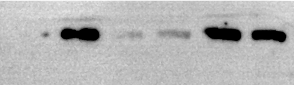

Supplement: Supplementary file 1 — Data S1. [file CNS-31-e70476-s001.zip › Supplemental Files/WB Figures/Full unedited gelblot for Figure 9d-P53/4 P53.tif]

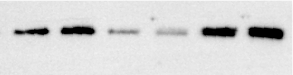

Supplement: Supplementary file 1 — Data S1. [file CNS-31-e70476-s001.zip › Supplemental Files/WB Figures/Full unedited gelblot for Figure 9d-P53/5 P53.tif]

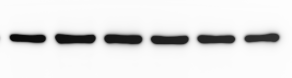

Supplement: Supplementary file 1 — Data S1. [file CNS-31-e70476-s001.zip › Supplemental Files/WB Figures/Full unedited gelblot for Figure 9d-β-actin/1 Full unedited gelblot for Figure 9d-β-actin.tif]

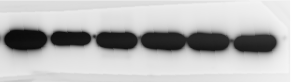

Supplement: Supplementary file 1 — Data S1. [file CNS-31-e70476-s001.zip › Supplemental Files/WB Figures/Full unedited gelblot for Figure 9d-β-actin/10 β-actin.tif]

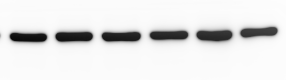

Supplement: Supplementary file 1 — Data S1. [file CNS-31-e70476-s001.zip › Supplemental Files/WB Figures/Full unedited gelblot for Figure 9d-β-actin/2 β-actin.tif]

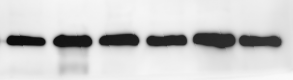

Supplement: Supplementary file 1 — Data S1. [file CNS-31-e70476-s001.zip › Supplemental Files/WB Figures/Full unedited gelblot for Figure 9d-β-actin/3 β-actin.tif]

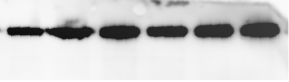

Supplement: Supplementary file 1 — Data S1. [file CNS-31-e70476-s001.zip › Supplemental Files/WB Figures/Full unedited gelblot for Figure 9d-β-actin/4 β-actin.tif]

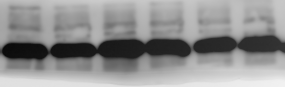

Supplement: Supplementary file 1 — Data S1. [file CNS-31-e70476-s001.zip › Supplemental Files/WB Figures/Full unedited gelblot for Figure 9d-β-actin/5 β-actin.tif]

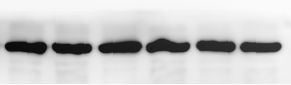

Supplement: Supplementary file 1 — Data S1. [file CNS-31-e70476-s001.zip › Supplemental Files/WB Figures/Full unedited gelblot for Figure 9d-β-actin/6 β-actin.tif]

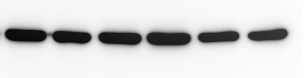

Supplement: Supplementary file 1 — Data S1. [file CNS-31-e70476-s001.zip › Supplemental Files/WB Figures/Full unedited gelblot for Figure 9d-β-actin/7 β-actin.tif]

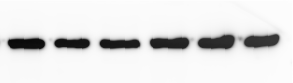

Supplement: Supplementary file 1 — Data S1. [file CNS-31-e70476-s001.zip › Supplemental Files/WB Figures/Full unedited gelblot for Figure 9d-β-actin/8 β-actin.tif]

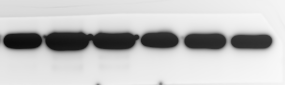

Supplement: Supplementary file 1 — Data S1. [file CNS-31-e70476-s001.zip › Supplemental Files/WB Figures/Full unedited gelblot for Figure 9d-β-actin/9 β-actin.tif]
